# First Ornithomimid (Theropoda, Ornithomimosauria) from the Upper Cretaceous Djadokhta Formation of Tögrögiin Shiree, Mongolia

**DOI:** 10.1038/s41598-017-05272-6

**Published:** 2017-07-19

**Authors:** Tsogtbaatar Chinzorig, Yoshitsugu Kobayashi, Khishigjav Tsogtbaatar, Philip J. Currie, Mahito Watabe, Rinchen Barsbold

**Affiliations:** 1 Department of Natural History Science, Graduate School of Science, Hokkaido University, Sapporo, 060-0810 Japan; 2 Hokkaido University Museum, Hokkaido University, Sapporo, 060-0810 Japan; 3 Division of Vertebrate Paleontology, Institute of Paleontology and Geology, Mongolian Academy of Sciences, Ulaanbaatar, 15160 Mongolia; 4 Biological Sciences, University of Alberta, Edmonton, T6G 2E9 Canada; 5 School of International Liberal Studies, Waseda University, Tokyo, 169-8050 Japan

**Keywords:** Palaeontology, Phylogenetics

## Abstract

The Upper Cretaceous Djadokhta Formation has been intensively surveyed for its fossil vertebrate fauna for nearly a century. Amongst other theropods, dromaeosaurids and parvicursorines are common in the formation, but ornithomimosaurs are extremely rare. A new ornithomimosaur material was discovered from the Djadokhta Formation, represented by eolian deposits, of the Tögrögiin Shiree locality, Mongolia. This is only the third ornithomimosaur specimen reported from this formation, and includes the astragalus, the calcaneum, the third distal tarsal, and a complete pes. The new material is clearly belonged to Ornithomimidae by its arctometatarsalian foot condition and has the following unique characters; unevenly developed pair of concavities of the third distal tarsal, curved contacts between the proximal ends of second and fourth metatarsals, the elongate fourth digit, and a laterally inclined medial condyle on phalanx IV-1. These diagnostic characters of the Djadokhtan ornithomimosaur indicate that this is a new taxon. Our phylogenetic analysis supports three clades within derived ornithomimosaurs, and the new taxon is placed a member of the derived ornithomimosaurs. The present specimen is the first ornithomimid record from eolian Tögrögiin Shiree locality, and is indicative of their capability to adapt to arid environments.

## Introduction

Ornithomimosauria, one of the major arctometatarsalian groups of non-avian dinosaurs, is a clade of highly specialized theropod dinosaurs which are characterized by edentulous jaw, long fore limb with unusual metacarpal proportions, and a powerful hind limbs. Since its first description is published, the diversity of ornithomimosaurs increased dramatically^1–6^.

Ornithomimosaurs are mainly known from the Cretaceous beds of Asia and North America, ranging from? Aptian-Albian to early Maastrichtian sediments^5,7^. The fossil occurrences of Asian ornithomimosaurs are rich in the Upper Cretaceous sediments, specifically from China and Mongolia^5^. Mongolian ornithomimosaurs are represented by five definitive taxa, *Anserimimus, Deinocheirus* and *Gallimimus* are from the Nemegt Formation (early Maastrichtian), *Garudimimus* is from the Bayanshiree Formation (Cenomanian-Santonian), and *Harpymimus* is from the Khukhteeg Formation (late Albian) formations^5^. Only two records of indeterminate ornithomimid specimens have been reported from the Campanian Djadokhta Formation at Ukhaa Tolgod^3,8^.

**Figure 1:**
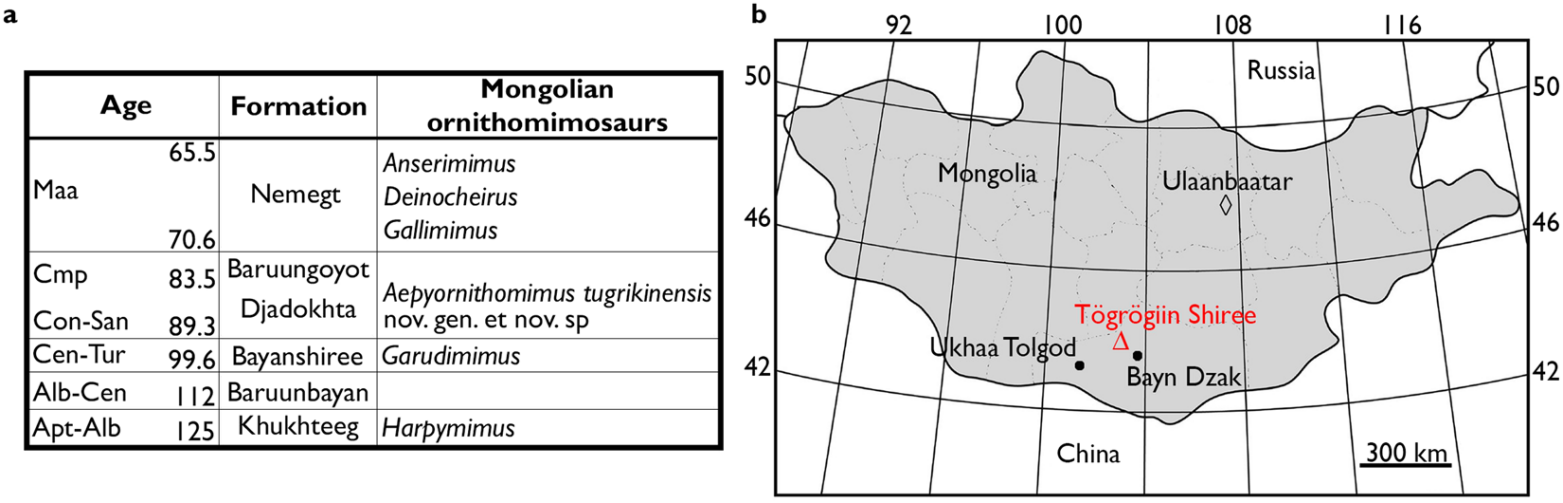
Location of *Aepyornithomimus tugrikinensis*. (**a**), Stratigraphic chart of ornithomimosaurs from Mongolia. (**b**), Location map. (◊), capital of Mongolia; (Δ), the position of type locality, Tögrögiin Shiree; (•), nearby other localities. Location map modified after Watabe *et al*.^65^.

The Djadokhta Formation has been intensively surveyed for its fossil vertebrate fauna for nearly a century^9^. Recent efforts continue to produce not only new specimens, but also new taxa^10,11^. The Djadokhta Formation unconformably overlies the Bayanshiree Formation (Cenomanian-Turonian) and is disconformably overlain by the Baruungoyot Formation (Santonian-Campanian)^12–15^. Although a physical contact between the two formations has not been fully identified^16^, the Djadokhta Formation is stratigraphically lower than the Baruungoyot Formation^14,17,18^ (Fig. 1a). The vertebrate assemblages of the Djadokhta Formation are rich in non-avian dinosaurs, such as dromaeosaurids^19^, mononychids^20^, troodontids^11^ and oviraptorids, ornithomimids are extremely rare^3,8,21^.

A new ornithomimosaur specimen was discovered from the Djadokhta Formation at Tögrögiin Shiree locality, about 50 km to the northwest of Bayn Dzak^17,22,23^ (Fig. 1b). The present specimen is the third ornithomimosaur record from this formation, and the first occurrence of Tögrögiin Shiree locality from Mongolia. This specimen is also the best preserved specimen of all of aforementioned three specimens known to date so far, and it provides new insight into ornithomimid dinosaur evolution and paleoenvironment.

## Results

### Systematic paleontology

Dinosauria Owen, 1842^24^.

Theropoda Marsh, 1881^25^.

Ornithomimosauria Barsbold, 1976^26^.

Ornithomimidae Marsh, 1890^27^.

*Aepyornithomimus tugrikinensis* gen. et sp. nov.

### Etymology

The generic name refers to the largest ratite bird *Aepyornis~*, which has similar pes structure; in Latin, ~*mimus* = ‘as’ or ‘like’; the species name *tugrikinensis* refers to the locality where the specimen was found.

### Holotype

**Figure 2:**
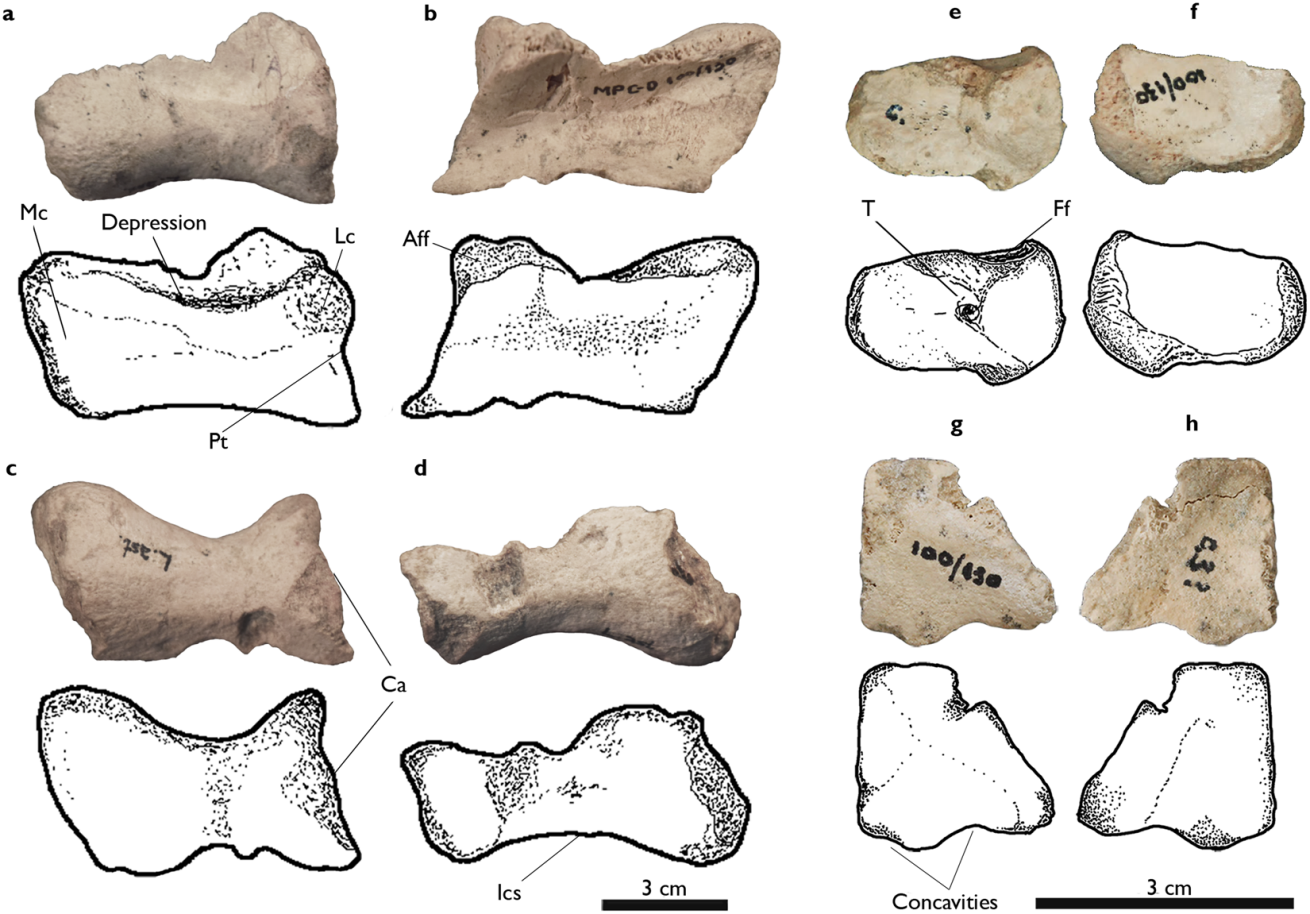
Ankle joint elements of *Aepyornithomimus tugrikinensis*. (**a–d**), the astragalus, (**e–f**), the calcaneum, and (**g–h**), the third distal tarsal, including line drawings. (**a**), in anterior, (**b,g**), in proximal, (**c,h**), in distal, (**d**), in posterior, (**e**), in medial, and (**f**), in lateral views. *Abbreviations:* (Aff), the anterior region of the fibular facet; (Ca), calcaneum insertion of the astragalus; (Ff), fibular facet; (Ics), intercondylar sulcus; (Lc), lateral condyle; (Mc), medial condyle; (Pt), protuberance; (T), tubercle.

**Figure 3:**
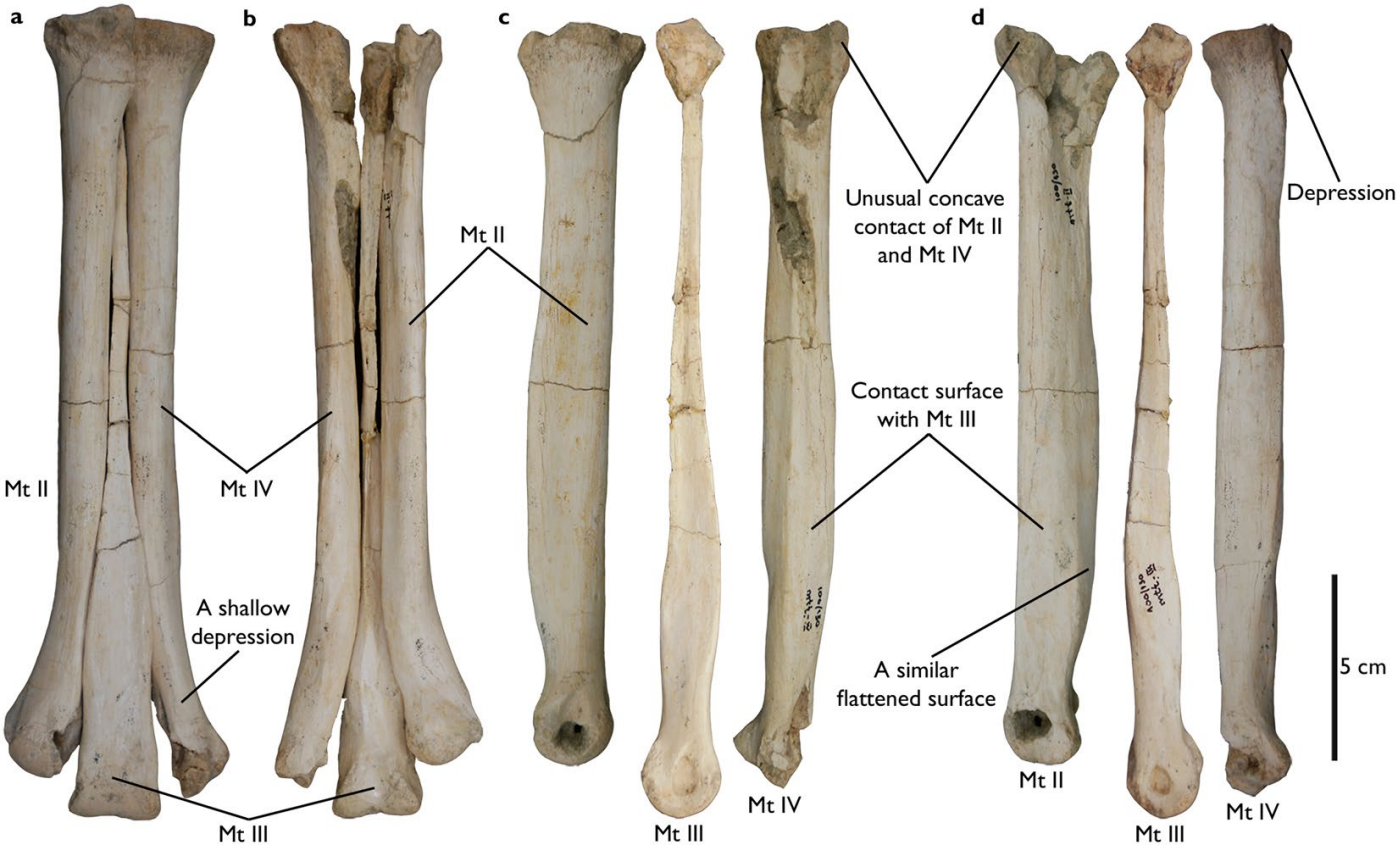
Metatarsals of *Aepyornithomimus tugrikinensis*. (**a**), in anterior, (**b**), in posterior, (**c**), in medial, and (**d**), in lateral views. *Abbreviations:* (Mt II, Mt III, and Mt IV), the second, the third, and the fourth metatarsals.

**Figure 4:**
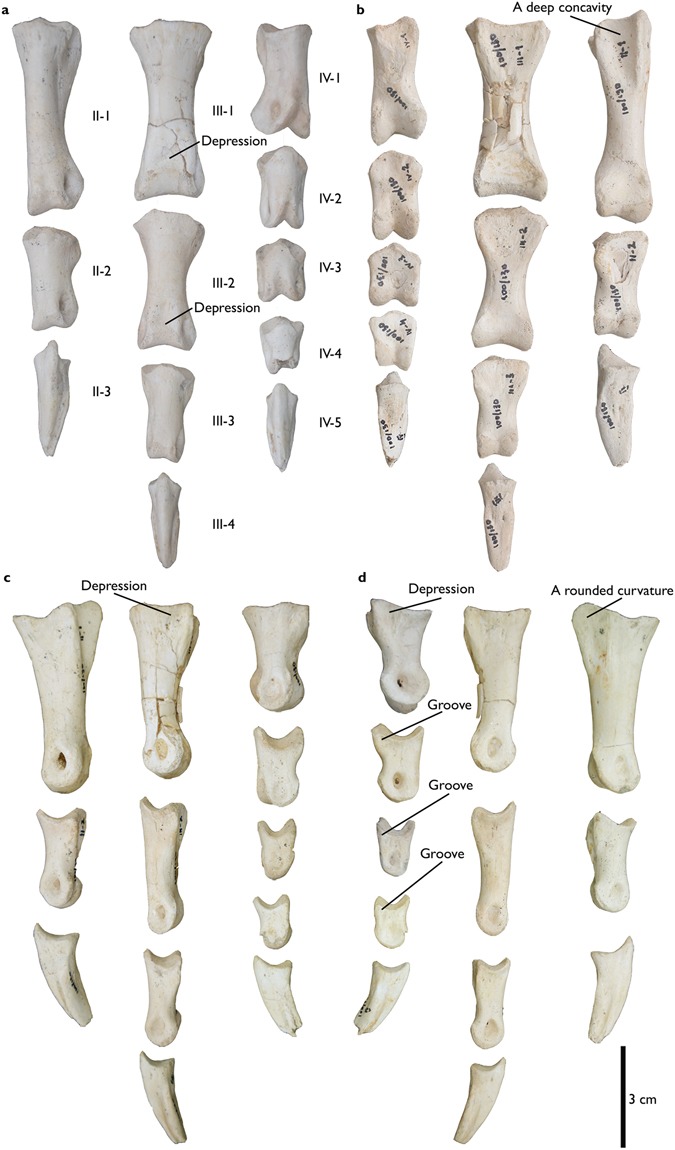
Phalanges of *Aepyornithomimus tugrikinensis*. (**a**), in dorsal, (**b**), in ventral, (**c**), in lateral, and (**d**), in medial views. *Abbreviations:* (II-1 to IV-5), phalangeal series of the second, the third, and the fourth digits.

MPC-D 100/130, articulated left pes preserved with an astragalus that is missing the ascending process, a complete calcaneum, and distal tarsal III (DT-III) (Figs 2, 3 and 4). The original specimen is now housed in the Institute of Paleontology and Geology of the Mongolian Academy of Sciences (IPG-MAS).

### Type locality and horizon

Central Sayr (44° 13′ 54″N, 103° 16′ 56″E) of Tögrögiin Shiree locality, Upper Cretaceous Djadokhta Formation (Campanian) (Fig. 1). This locality is interpreted as semi-arid eolian sediments^28^ with up to 52 m of light gray, cross-bedded, structureless sands and sandstones^17^.

### Diagnosis

An ornithomimid dinosaur with the following unique characters; unevenly developed pair concavities on the posterior margin of the DT-III; robust distal articular caput of second metatarsal (Mt II) in dorsal view; proximoventrally rounded ridge of phalanx II-1 (II-1); the elongate fourth digit; laterally inclined medial condyle of phalanx IV-1 (IV-1); elongated pedal unguals.

## Description

*Tarsus*. Preserved tarsal bones include the astragalus, calcaneum and DT-III. Most of these elements are complete, although the ascending process of the astragalus is missing. The astragalus and calcaneum are not fused. The distal condyles of the astragalus are unevenly developed so that the medial condyle is more pronounced than the lateral one. It leans more anteromedially than in Early Cretaceous taxa^6^ *Gallimimus, Qiupalong* and *Struthiomimus*^29–31^ (Fig. 2c). A depression is present at the base of the ascending process in anterior view (Fig. 2a). The intercondylar sulcus is deeply concave as in *Garudimimus* and *Harpymimus*, but unlike more derived ornithomimosaurs in which it is shallow^29,32^ (Fig. 2a,d). The outer margin of the lateral condyle is notched for receiving the medial tubercle of the calcaneum, as in other ornithomimosaurs (Fig. 2a,c). However, the notch of *Aepyornithomimus tugrikinensis* is not as deep as some taxa of derived ornithomimosaurs^29^.

The calcaneum is a thin and disc-like bone (Fig. 2e,f). It extends anteroposteriorly to make an oval shape. It differs from most ornithomimosaurs by shape where is often represented as a round. A weakly developed tubercle is positioned at the center area (Fig. 2e). The facet at the proximal surface indicates that the fibula contacted the tarsus (Fig. 2e). The lateral surface is flat, but slightly concave as like those *Gallimimus* and *Garudimimus*^30,32^ (Fig. 2f).

One of the distal tarsals was preserved in the specimen. It supposed the distal tarsal III. It is a nearly complete, however part of the anteromedial edge is missing (Fig. 2g,h). DT-III is a thin proximodistally. Although the proximal end of Mt III is missing, DT-III would have been covered the proximal articular surface of Mt III completely or partially. It also partially covered the proximal end of Mt II as in other ornithomimosaurs. DT-III has developed a pair of uneven concavities on the posterior edge (Fig. 2g,h). The medial concavity is deeper than lateral one. The corresponding edge of DT-III is almost straight in *Garudimimus*, but is convex in *Gallimimus* and the Bissekty ornithomimid^33–36^. Moreover, the overall shape of DT-III is triangular with a straight medial edge in proximal view, whereas it is quadrangular with a convex medial edge in other ornithomimosaurs.

### Metatarsals

Mt II, Mt III, and Mt IV are preserved. Metatarsals are generally complete, but are missing some parts of the distal end of Mt II, the proximal end of Mt III, and the medial condyle of Mt IV (Fig. 3). Mt I is not preserved and no any sense of Mt I in the metatarsals. The length ratios are as usual in ornithomimosaur, like Mt III is the longest, and Mt II is marginally shorter (98%) than Mt IV (Fig. 3a).

The third digit of *Aepyornithomimus tugrikinensis* is shorter than those Late Cretaceous ornithomimosaurs when comparing to the total length of Mt III with the third digit length. However, this length is a comparable to *Deinocheirus* and *Garudimimus*^4,32^. The metatarsals are more slender than Deinocheiridae; in this sense, the relatively slender metatarsals of *Aepyornithomimus tugrikinensis* are more resembled as derived ornithomimosaurs by its presence^2,29,30,34^.

**Figure 5:**
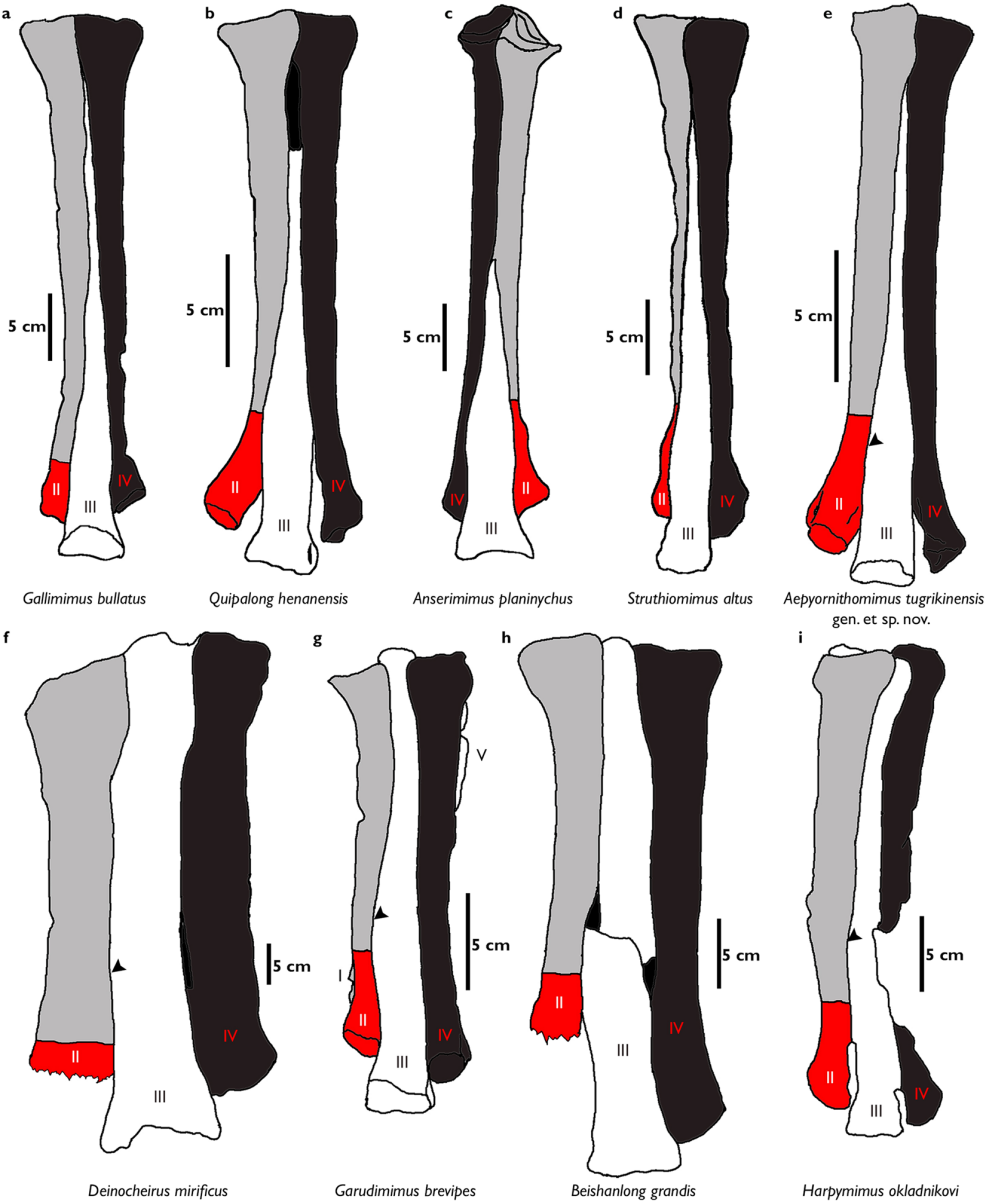
Comparisons of ornithomimosaur metatarsals. (**a–e**), “arctometatarsalian” condition, (**f**–**i**), “non-arctometatarsalian” condition, *Abbreviations:* (II), the second metatarsal with comparison of the distal expansion, (III), the third metatarsal, and (IV), the fourth metatarsal, and (▶), degrees of the medial expansion of each taxon (Supplementary Table S2).

The lengths of Mt II and Mt IV are subequal in *Aepyornithomimus tugrikinensis*, like *Anserimimus*, *Gallimimus*, and *Qiupalong*^37^, whereas Mt IV is longer than Mt II in most ornithomimosaurs (Fig. 5). The outlines of the proximal articular surfaces of Mt II and Mt IV of *Aepyornithomimus tugrikinensis* resemble *Quipalong*^31^. In some taxa, these metatarsals tightly adhere to Mt III distally, whereas metatarsals of other taxa are distinctly divergent^5^. The distal articular surfaces are rounded as in other ornithomimosaurs^5^. The collateral ligament fossae of Mt II and Mt IV are equally developed with the same depths.

**Figure 6:**
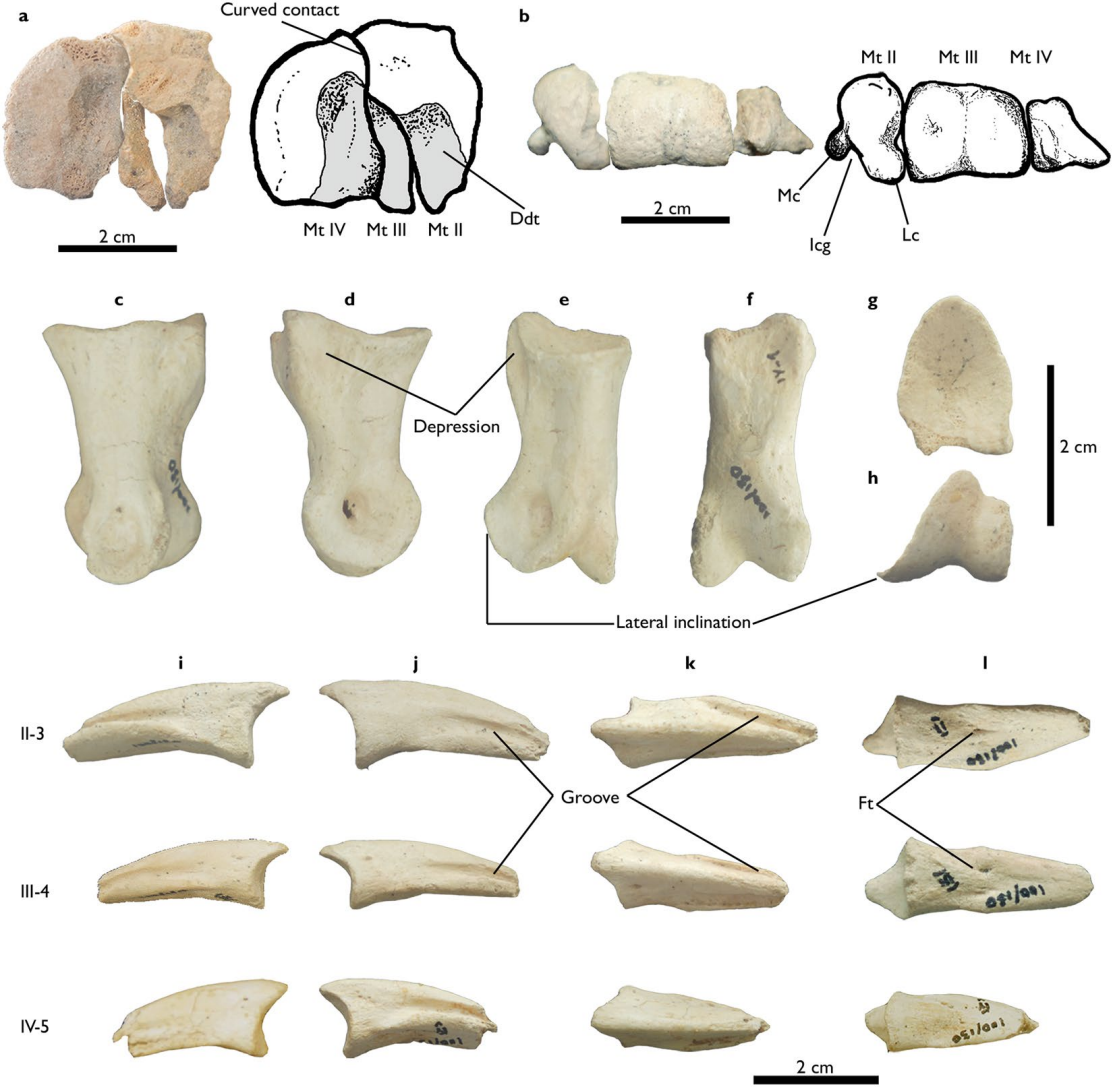
The articulated views of metatarsals, the first phalanx (IV-1) of digit IV, and the ungual phalanges of *Aepyornithomimus tugrikinensis*. (**a**), in proximal end, and (**b**), in distal end of metatarsals. (**c**–**l**), Phalanges IV-1, II-3, III-4, and IV-5 – (**c**,**i**), in lateral, (**d**,**j**), in medial, (**e**,**k**), in dorsal, (**f**,**l**), in ventral, (**g**), in proximal, and (**h**), in distal views. *Abbreviations:* (Ddt), facet of the distal tarsals; (Ft), flexor tubercle; (Icg), intercondylar groove; (Lc), lateral condyle; (Mc), medial condyle; (Mt II, Mt III, Mt IV), the second, the third, and the fourth metatarsals; (II-3, III-4, IV-5), ungual phalanges of the digits.

Mt II is nearly complete, but the lateral sides of both proximal and distal ends are crushed naturally (Fig. 3d). A morphologically interesting feature is present on the proximal end of metatarsus. The anterolateral surface of the proximal end of Mt II has a deep, rounded concavity for receiving the convex anteromedial surface of Mt IV; it forms an unusual curved contact in proximal view (Figs 3d and 6a). This contact is straight in other ornithomimosaurs, such as *Anserimimus, Gallimimus, Ornithomimus*, and Ornithomimid indet. (MPC-D 100/14)^5,34,38^. The shaft of the diaphysis is straight and slender. The cross-section of Mt II is presumably subcircular. The width of the distal articular end is nearly the same as the width of the distal articulation of Mt III, which is unusual in ornithomimosaurs (Fig. 3a). The distal fifth of Mt II diverges medially from Mt III, but the degree of divergence is less than in *Anserimimus* and *Gallimimus*^29,34^. Its divergence is relatively greater than in basal ornithomimosaurs^32,36,37^, although it is similar to the type specimen of *Qiupalong*^31^. The lateral condyle of the Mt II is larger than the medial condyle, and these condyles are separated by a deep sulcus on the flexor side (Fig. 6b), which is even deeper than in *Anserimimus, Harpymimus*, and IVPP V12756^34,36,39^. The lateral collateral ligament fossa is somewhat weathered but visible.

Mt III is the longest metatarsal of other metatarsals (Fig. 3). Based on the configuration of the proximal contacts of Mt II and Mt IV, the proximal end of Mt III seems to be a triangular and tapering posteriorly (Fig. 6a). The proximal end is pinched as like *Gallimimus* and *Struthiomimus*. However, it differs from *Anserimimus*, in which the proximal half of the Mt III shaft is completely covered by other metatarsals (Fig. 5c). The anteromedial edge is straighter than the anterolateral edge (Fig. 3a). This feature is similar to one of diagnostic characters of *Rativates*^40^. The cross-section of the distal half of Mt III is subrectangular. Mt III broadens distally and slightly covers the lateral and medial edges of Mt II and Mt IV, respectively^41,42^. The anterolateral margin of MT III is more widely separated from Mt IV than it is from Mt II (Fig. 3a), whereas both margins deviate equally in tyrannosaurids^43^. There is a shallow depression on the anterior surface of the distal half of Mt III (Fig. 3a). The distal end of Mt III is similar to those ornithomimosaurs^5^. However, the medial condyle is slightly larger than the lateral condyle^29,32,37,39^. The distal intercondylar groove is shallower than those of the Mt II and Mt IV. The articular caput of Mt III is as straight as *Rativates* on the flexor side (Fig. 6b)^40^.

The nearly complete Mt IV is fractured in the proximomedial and distal parts (Fig. 3c,d). The medial and posterior surfaces of the proximal two-thirds of the shaft are flat (Fig. 3b,c). The shaft is oval to rectilinear in cross-section, and its width is less than its anteroposterior length. Although the fifth metatarsal is not preserved in *Aepyornithomimus tugrikinensis*, there is a deep depression to receive it on the proximolateral surface of Mt IV^4,32^ (Fig. 3d).

### Pedal phalanges

All pedal phalanges (II-1 to II-3, III-1 to III-4, and IV-1 to IV-5) are preserved (Fig. 4). The phalangeal formula is 0-3-4-5-0 as in other ornithomimosaurs, except for *Beishanlong*^6^ and *Garudimimus*^32^, both of which retained the first digit. Although the second and the fourth digits are shorter than the third digit as typical, the corresponding digits are relatively longer than in other ornithomimosaurs. The proximal halves of the ventral surfaces of all phalanges are flat, with the exception of II-1.

Articular surfaces of the most proximal phalanges (II-1, III-1, and IV-1) are shallow, undivided concavities. II-1 and III-1 are the most robust of the phalangeal series, although II-1 is slightly longer than III-1. The heights of the proximal ends of II-1 and IV-1 are greater than their widths, whereas the relationship is the opposite in III-1. There are shallow depressions on the lateral surfaces of II-1 and III-1, and a slightly stronger depression on the proximomedial surface of IV-1 near the proximoventral end (Figs 4c,d and 6d,e). The proximoventral surface of II-1 has a deep concavity like other derived Asian ornithomimosaurs, but unlike that of *Tototlmimus*^35^. Also, the proximomedial boundary forms a rounded curvature (Fig. 4d). It is different than other ornithomimosaurs, especially *Garudimimus*, and *Tototlmimus*, where the corresponding boundary is rectilinear^29,35,37,44^.

The proximal articular surface of II-2 is divided by a low vertical ridge for its weak ginglymoid joint with II-1. The lip-like proximodorsal process of II-2 overlaps its joint with II-1 (Fig. 4a). The length ratio of II-1 to II-2 is approximately 2:1. The length of II-2 is subequal to IV-1.

III-1 and III-2 are similar in appearance, although the lengths are different. The proximal articular surfaces of the third digit phalanges (III-1 to III-3) are undivided and nearly symmetrical, whereas there are strong, vertical ridges on the proximal articular surfaces of IV-2 to IV-4. The medial condyle of III-1 is slightly extended to distally. There are faint depressions on the dorsal surfaces of III-1 and III-2. The distal articular surface of III-3 is asymmetrical, and its lateral condyle is larger than the medial one in dorsal view. In addition, deep short grooves are presented along the proximomedial surfaces of IV-2 to IV-4, which are similar to those seen in III-1 and III-2 of *Garudimimus*^32^ (Fig. 4d).

Successive phalanges of the fourth digit of *Aepyornithomimus tugrikinensis* become mediolaterally slender (Table 1 and Fig. 4)^2,29,30^. Like phalanges of the second digit, those of the fourth digit have weakly ginglymoid joints. The lateral condyle of IV-1 is a relatively smaller (Fig. 6h). Its medial condyle is inclined more laterally than in any other ornithomimosaurs. The degree of inclination is about 35°, when it is measured from the base of the medial condyle of the IV-1 to the lateral. This degree is less in other ornithomimosaurs (e.g., *Deinocheirus* 21°, *Harpymimus* 17°, and *Struthiomimus* 25°). Phalanx IV-1 has a deep depression dorsal to the proximoventral ridge in medial view. Phalanx IV-3 is somewhat unlike other ornithomimosaurs in having a flattened flexor surface, a deeper proximal concavity, and a more elongate appearance in lateral and medial views. The medial collateral ligament pits of IV-1 to IV-4 are deeper than the corresponding lateral ones. However, the lateral ones are still deeper than their equivalents in *Deinocheirus*^4^ and *Harpymimus*^37^ (Fig. 4c,d).

**Table 1:** Measurements (in mm) of *Aepyornithomimus tugrikinensis*, (MPC-D 100/130). *Explanation:* (−), missing elements, (+), incomplete elements.

Elements	Length	Proximal end		Distal end
		width	height	width
Astragalus	51.8	—	—	—
Calcaneum	25.06	—	—	—
Metatarsal II (Mt II)	201	19.19	36.7	21.61
Metatarsal III (Mt III)	211	5.69	18.22+	25.15
Metatarsal IV (Mt IV)	207	19.23	25.26	16.64+
Phalanx II-1 (II-1)	59.08	17.87	24.07	17.07
Phalanx II-2 (II-2)	32.09	16.99	14.94	14.30
Phalanx II-3, ungual (II-3)	36.35+	11.10	13.49	—
Phalanx III-1 (III-1)	52.11	24.84	21.34	21.54
Phalanx III-2 (III-2)	42.73	21.84	13.80	18.25
Phalanx III-3 (III-3)	29.48	16.80	11.94	12.12
Phalanx III-4, ungual (III-4)	30.89+	10.86	10.88	—
Phalanx IV-1 (IV-1)	32.88	15	20.89	18.16
Phalanx IV-2 (IV-2)	24.34	15.58	15.67	14.04
Phalanx IV-3 (IV-3)	19.80	15.32	11.76	14.66
Phalanx IV-4 (IV-4)	17.68	13.56	10.95	10.62
Phalanx IV-5, ungual (IV-5)	27.74+	10.05	12.38	—

All unguals are triangular in cross-section, but mediolaterally more slender than other ornithomimosaurs (Fig. 6i–l). The articular surfaces of ungual phalanges II-3 and IV-5 are asymmetric to match the distal ends of the penultimate phalanges. In contrast, the ungual phalanx III-4 is symmetrical to the dorsoventral axis. Flexor tubercles are weakly developed or almost not existed in all unguals, similar to *Qiupalong*^31^ and *Tototlmimus*^35^, but different from the Bissekty ornithomimid^44^ (Fig. 6l). All unguals have shallow lateral and medial grooves, extending from the proximal articular edges to the distal tips (Fig. 6i,j). In addition, sulci do not exist along the ventromedial and ventrolateral edges of any unguals known for *Struthiomimus*^35^ and *Tototlmimus*^35^. On the other hand, the general appearances of the shallow sulci and the proximodistally elongate unguals are similar to those of *Nqwebasaurus*^45^. Ungual phalanx II-3 of *Aepyornithomimus tugrikinensis* is relatively larger than the other two unguals (Fig. 6i–l). Unguals of the second and the fourth digits are inclined somewhat outward from their inner parts as in most ornithomimosaurs. Similar to *Archaeornithomimus*, *Beishanlong* and *Qiupalong*, the ventral surfaces of the unguals are slightly curved ventrally in lateral view^5,30,31^, but the condition of unguals in *Ornithomimus* and *Tototlmimus* is a nearly straight which are differentiated *Aepyornithomimus tugrikinensis* from these taxa.

### Phylogenetic analysis

**Figure 7:**
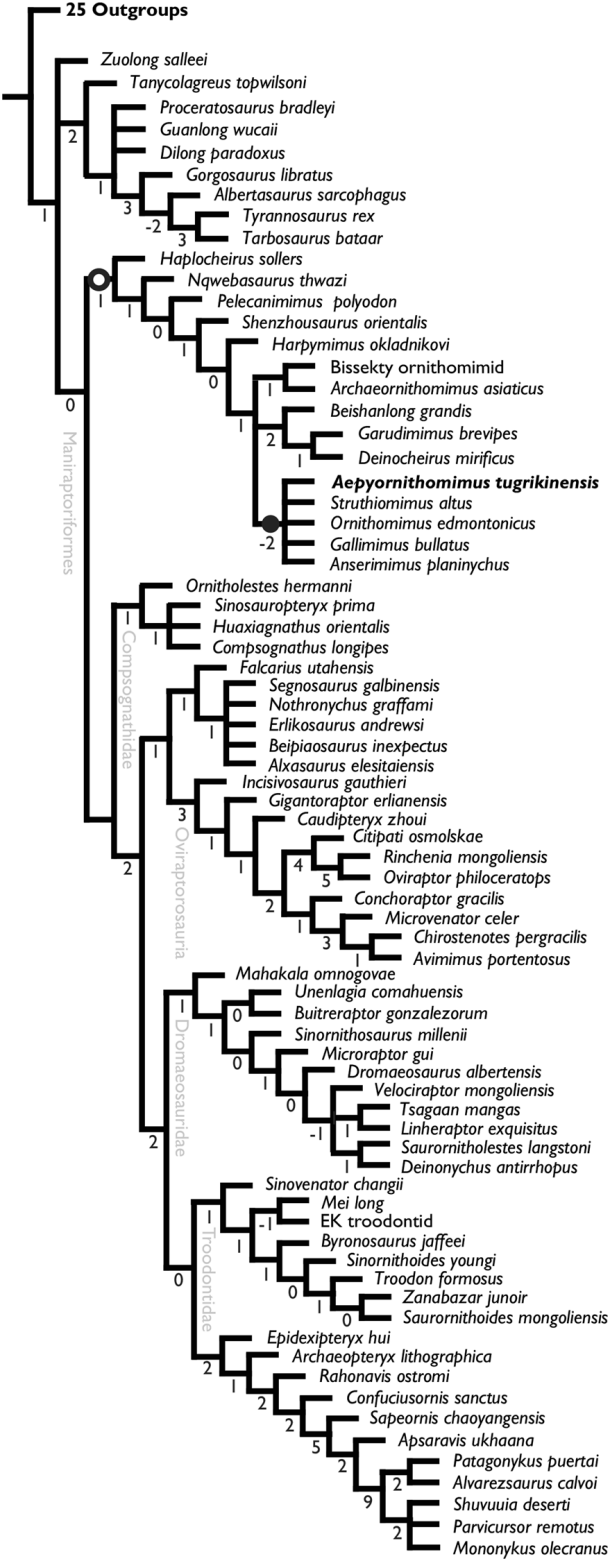
Strict consensus tree of the phylogenetic relationships of *Aepyornithomimus tugrikinensis* within the Coelurosauria. *Explanation:* (-I, -2….9), Bremer supports, (○), Ornithomimosauria, and (∙), Ornithomimidae.

In order to assess the phylogenetic position of *Aepyornithomimus tugrikinensis*, this taxon was added to a recently published modified dataset of coelurosaurians^4,44,45^. Our analysis recovers four most parsimonious trees (MPTs) (Supplementary Fig. S1). The strict consensus tree shows that basal ornithomimosaurs, from *Haplocheirus* to *Harpymimus*, are successive taxa, the monophyly of three clades of derived ornithomimosaurs (Deinocheiridae, *Archaeornithomimus* + Bissekty ornithomimid, and the clade of *Anserimimus*, *Aepyornithomimus tugrikinensis, Gallimimus*, *Struthiomimus*, and *Ornithomimus* (called “derived ornithomimids” herein)) (Fig. 7). The relationships of Deinocheiridae, *Archaeornithomimus* + Bissekty ornithomimid, and “derived ornithomimids” remain an unresolved polytomy.

The clade of “derived ornithomimids” is well-supported by sharing the following unambiguous synapomorphic characters; the maxillary fenestra is recessed within a posteriorly shallow recessed maxillary fenestra **[17]**, a descending process of the squamosal is parallel to the shaft of the quadrate **[110]**, the quadrate is hollow **[112]**, scapula is longer than humerus **[361]**, absent or poorly developed medial tab on the proximal end of Mt I **[395]**, more or less symmetrical condyles of the Mt I **[396]**, the supraacetabular crest forms a hood over the femoral head **[448]**, the shaft of Mt IV is round or thicker than wide in cross-section **[558]**, and the pedal unguals have pronounced flexor fossae on the ventral surfaces of the proximal ends **[567]**.

There are two potential relationships among Deinocheiridae, *Archaeornithomimus* + Bissekty ornithomimid, and the clade of “derived ornithomimids” (Supplementary Fig. S1). Two of four MPTs show two clades, Deinocheiridae and Ornithomimidae (*Archaeornithomimus*, Bissekty ornithomimid, and “derived ornithomimids”), as suggested by previous studies^4^. The clade of Ornithomimidae shares following three unambiguous synapomorphies, absence of posterolateral crests on lateral surfaces of cervical centra **[262]**, completely closed pubic apron **[463]**, and smooth and not ginglymoid distal end of Mt III **[553]** (Supplementary Fig. S1a,b). The other two MPTs suggest Deinocheiridae and “derived ornithomimids”, are monophyletic, supported by two synapomorphies; the parapophysis is distinctly below the transverse process in the most posterior dorsal vertebrae **[299]** and the ulnar shaft is straight **[375]** (Supplementary Fig. S1c,d). The change in the phylogenetic position of *Archaeornithomimus* + the Bissekty ornithomimid is probably related to the fragmentary nature of these specimens.

The monophyletic “derived ornithomimids” suggests that *Aepyornithomimus tugrikinensis* belongs to Ornithomimidae, although interrelationships among these taxa are poorly resolved (Fig. 7). This clade is supported in all MPTs by sharing three unambiguous synapomorphies; absent or poorly developed medial tab on the proximal end of Mt I **[395]**, more or less symmetrical condyles of Mt I **[396]**, and the supraacetabular crest forms a hood over the femoral head **[448]**. *Anserimimus* is placed as the most basal taxon in this clade, and each MPT shows different phylogenetic positions for *Aepyornithomimus tugrikinensis*. MPTs 1, 2, and 4 support the monophyly of *Aepyornithomimus, Gallimimus, Ornithomimus*, and *Struthiomimus* with two unambiguous synapomorphies (scapula longer than humerus **[361]** and penultimate phalanx of the second digit longer than first phalanx **[411]**) although *Aepyornithomimus* does not preserve forelimbs. In MPTs 1 and 4, *Aepyornithomimus tugrikinensis* is nested together with North American taxa (*Ornithomimus* and *Struthiomimus*) and shares the following synapomorphies; posterodorsal process of the lacrimal projects posterodorsally **[78]**, articular has elongate, slender medial, posteromedial, or mediodorsal process from retroarticular process **[208]**, and postzygapophyses of cervical vertebrae 2–4 are connected medially along their entire lengths by a dorsally concave intrazygapophyseal lamina for attachment of interspinous ligaments **[271]** (Supplementary Fig. S1a,d). These alternative positions of *Aepyornithomimus tugrikinensis* are due to its preservation in nature, and additional materials are required to determine the more obvious position of this taxon in the future.

## Discussion

*Aepyornithomimus tugrikinensis* is the first ornithomimid ornithomimosaur identified from the Upper Cretaceous Djadokhta Formation of Tögrögiin Shiree in Mongolia and Late Cretaceous new member of this clade that has been named from Mongolia after nearly three decades^14,42^. Previously, only two ornithomimosaur materials have been reported from the Ukhaa Tolgod locality^3,8^. Insufficient materials in nature, these specimens were unable to display any diagnostic characters for determining their taxonomic certainty. In addition, no any overlapped elements between *Aepyornithomimus tugrikinensis* and Ukhaa Tolgod materials that are not permit to compare them in this study.

*Aepyornithomimus tugrikinensis* has some unique features on the foot. For instance, it differs from *Beishanlong, Deinocheirus, Garudimimus*, and *Harpymimus* in having relatively slender “arctometatarsalian” metatarsals^4,6,32,36,37,46^, the unusual curved contact between the proximal ends of Mt II and Mt IV, and a robust distal articular caput of Mt II. In contrary to, all other Late Cretaceous ornithomimosaurs have a straight Mt II and Mt IV contact at the proximal end and a relatively small distal articular caput of Mt II^47^.

**Figure 8:**
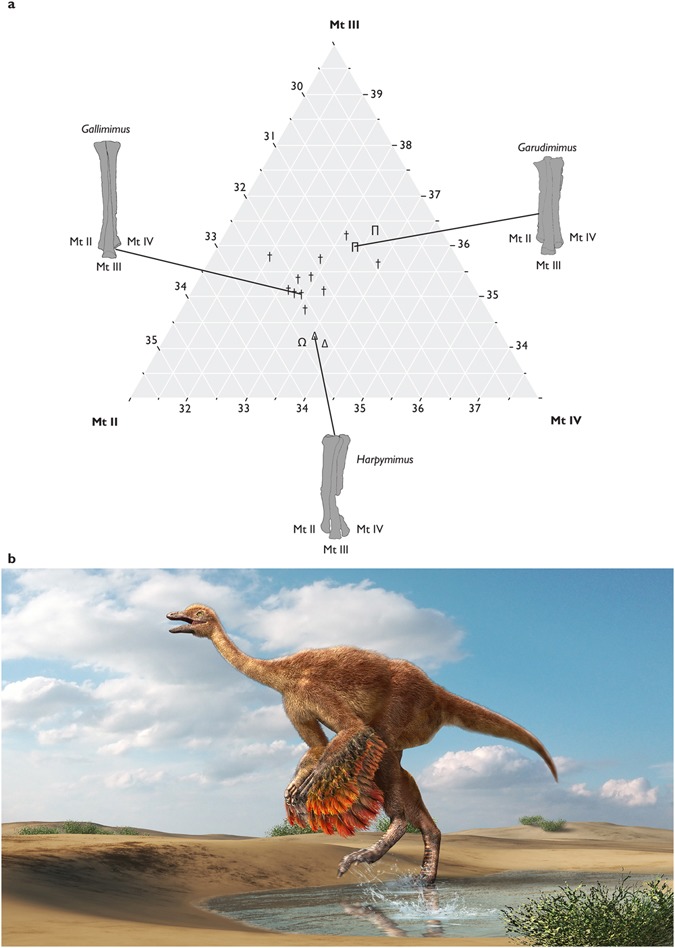
Comparative graph and restoration drawing of *Aepyornithomimus tugrikinensis*. (**a**), Different proportions of the three metatarsals is represented by ternary diagram (Supplementary Table S1), (**b**), Illustration is drawn by Mr. Masato Hattori. *Abbreviations:* (Mt II), the metatarsal II, (Mt III), the metatarsal III, and (Mt III), the metatarsal III, (Ω), *Aepyornithomimus tugrikinensis*, (Δ), basal ornithomimosaurs, (Π), deinocheirids, (†), ornithomimids.

The proportional differences of three metatarsal elements are compared among ornithomimosaur species by a ternary diagram (Fig. 8a and Supplementary Table S1). Individual metatarsal measurements are averaged for each species. The diagram shows that basal ornithomimosaurs (*Nqwebasaurus* and *Harpymimus*), deinocheirids, and ornithomimids bear generally different metatarsal proportions. In addition, basal ornithomimosaurs have shorter Mt III, whereas deinocheirids have shorter Mt II. *Aepyornithomimus tugrikinensis* shares a similar metatarsal proportion with basal ornithomimosaurs. A discriminant analysis is used three categorized groups (basal ornithomimosaurs, Deinocheirids, and Ornithomimids). Mt II, Mt III, and Mt IV lengths as covariates that confirm the morphological separation among the three groups (Wilk’s lambda = 0.321, *F* = 2.803, *p* = 0.03). Twelve species out of sixteen species are correctly classified in the analysis. *Aepyornithomimus tugrikinensis* is classified into basal ornithomimosaurs with posterior probability of 0.858.

The mediolaterally slender phalanges of the fourth digit and the laterally inclined medial condyle of the IV-1 are unique characters of *Aepyornithomimus tugrikinensis*. In contrary to the derived ornithomimosaurs, the phalangeal lengths of the fourth digit are also long in basal ornithomimosaurs, like *Garudimimus* and *Harpymimus*. In spite of this, the elongate fourth digit of *Aepyornithomimus tugrikinensis* is distinct from those of derived ornithomimosaurs, such as *Anserimimus, Gallimimus, Ornithomimus*, and *Struthiomimus*. The phalanges of these taxa are highly abbreviated^29,30,34^. The degree of inclination of the medial condyle of IV-1 is 35° which is greater than any ornithomimosaurs.

In addition, the length from the distal end of Mt III to its medial expansion is scored as 0.161 in *Aepyornithomimus tugrikinensis*, which is the lowest value for any ornithomimosaur taxa. This feature indicates *Aepyornithomimus tugrikinensis* is closer to derived ornithomimosaurs than to basal ornithomimosaurs, which confirms an intermediate step towards an arctometatarsalian condition of Ornithomimosauria as suggested by Currie^48^ (Fig. 5e–g,i and Supplementary Table S2).

Besides these characters, *Aepyornithomimus tugrikinensis* is differentiated all other ornithomimosaurs by following characters; unevenly developed pair of concavities present at posterior edge of the DT-III of *Aepyornithomimus tugrikinensis*. This morphology is different from any other ornithomimosaurs where posterior edge is either convex or concave^32–35^; the proximoventral ridge of II-1 is round, II-3 is relatively larger than the other two unguals, and the pedal unguals are anteroposteriorly more slender and curve slightly downward.

The foot of *Aepyornithomimus tugrikinensis* is also compared with local fauna of the Djadokhta Formation which are persisted the same condition. The foot of *Aepyornithomimus tugrikinensis* is similar to *Avimimus portentosus* and *Kol ghuva* by the “arctometatarsalian” condition, and the subequal length of Mt II and Mt IV^49–51^. However, some characters of both *Avimimus* and *Kol* are differentiated *Aepyornithomimus tugrikinensis*. Whereas a co-ossified tarsometatarsus, a short phalanx II-2 than the phalanx II-1, and the abbreviated phalanges of Digit IV are characteristics of *Avimimus*, the alvarezsaurids *Kol* is differentiated by following features, such as almost no sign of the Mt III from the posterior view, and a presence of the first digit^49,52^.

Metatarsals are the most common recovered elements among troodontids from the Djadokhta Formation^53^. The condition of the asymmetrical metatarsals of *Gobivenator mongoliensis*^12^ is clearly distinguished from any ornithomimids. Whereas the lengths of Mt II and Mt IV of *Aepyornithomimus tugrikinensis* are subequal in length, Mt II of all known troodontids is mediolaterally compressed and markedly shorter than the more robust Mt IV^54^. The phalanges of Digit II are also highly modified in troodontids^47,55^.

### Palaeobiogeography

The increasing number of fossil discoveries suggests that ornithomimosaurs were highly diverse during the Late Cretaceous of central Asia and western North America^5^. However, the origin of ornithomimosaurs is poorly understood, particularly when it comes to more basal members discovered in Europe and Africa^45,56^. Until now, seventeen valid species, belonged to sixteen genera, have been assigned to Ornithomimosauria. Among derived ornithomimosaurs, their fossil occurrences are concentrated in the Late Cretaceous (Campanian to Maastrichtian) of Asia and North America. North American derived ornithomimosaurs (*Ornithomimus, Rativates*, and *Struthiomimus*) have been mainly recovered from the Campanian, indicative of a diversity of ornithomimosaurs in this time bin. In contrast, derived ornithomimosaurs (*Anserimimus, Deinocheirus*, and *Gallimimus*) in Mongolia seem to be more diverse in the Maastrichtian^48^. However, Campanian ornithomimosaur is extremely rare in Mongolia^30^.

A complex palaeobiogeographic aspect involving multiple dispersals between Asia and North America that has been supported for several Late Cretaceous dinosaur clades, including hadrosauroids, dromaeosaurids^10,57^ Makovicky and his colleagues are proposed that the derived monophyletic clade, *Struthiomimus* and *Ornithomimus*, from North America accounted for at least one dispersal event from Asia to North America across Beringia^5^. Alternatively, one more dispersal event probably occurred into North America or a back to Asia if *Ornithomimus* and *Struthiomimus* are not immediate sister taxa^5^. In addition, one widely accepted palaeobiogeographic analyses of ornithomimosaurs is suggested by Kobayashi and Barsbold that supported an Asian origin for Ornithomimidae and had occurred a single dispersal events of ornithomimids from Asia into North America during, or prior to, the Campanian^58^.

Based on patterns of the most parsimonious trees of the phylogeny, dispersal event of Ornithomimidae was probably occurred in two directions (from Asia to North America and back to Asia from North America) across Beringia in during Late Cretaceous (Supplementary Fig. S1a,b). Tree topologies suggest two possibilities. The second and the third patterns show a monophyly of North American taxa (*Ornithomimus* and *Struthiomimus*), suggesting a single dispersal by the Campanian (Supplementary Fig. S1b,c). Two dispersal events with two scenarios are implied by the first and the fourth patterns because *Aepyornithomimus tugrikinensis* is nested together with North American taxa (*Ornithomimus* and *Struthiomimus*) and *Struthiomimus* is a sister taxon to *Aepyornithomimus tugrikinensis* (Supplementary Fig. S1a,d). One scenario is that the first dispersal event was occurred from Asia to North America during Campanian time, and the second dispersal event was returned from North America to Asia by *Aepyornithomimus tugrikinensis*. The other scenario is two independent dispersals (*Ornithomimus* and *Struthiomimus*) from Asia to North America during the Campanian.

### Paleoenvironment and Paleoecology

During the Late Cretaceous, the vertebrates of Mongolia lived in different climatic conditions than the contemporaneous continental North American faunas^59^. The Late Cretaceous paleoenvironments of the major dinosaur-bearing formations of the Gobi Desert are divided into three main successions (Baruungoyot, Djadokhta, and Nemegt formations).

While perennial, widespread lacustrine sedimentation were predominated in the Lower Cretaceous and early Late Cretaceous (the Bayanshiree Formation), the Campanian Djadokhta Formation and subsequent units were mainly dry conditions, characterized by eolian-influenced environments with a lacking permanent fluvial drainage system^14^. Whereas the Djadokhta Formation has been documented as primarily arid with minor ‘wet’ facies, which was influenced by large lakes^59^, the Maastrichtian Nemegt Formation has been interpreted as a mostly fluvial environment with most fossils from channel fill, point bar, and occasional overbank deposits laid down under more humid condition^12^. Nonetheless, part of the Nemegt Formation is time equivalent to the Baruungoyot Formation, and more specifically to dry, eolian deposits of this Formation. Some of dinosaur groups such as troodontids, dromaeosaurids, oviraptorids, hadrosaurids, and birds are well-known in both Djadokhta and Nemegt formations, suggesting that members of these dinosaur groups were adapted for both arid and wet environments^10,11,17,59^. Furthermore, the paleoenvironment conditions and vertebrate faunas are represented mostly similar in both Baruungoyot and Djadokhta formations, none of ornithomimosaur specimens haven’t yet been reported from the Baruungoyot Formation in Mongolia^5,14^. *Aepyornithomimus tugrikinensis* is possibly the first evidence of an ornithomimosaur taxon that could have tolerated more diverse climatic conditions that were shifting from humid to more arid conditions (Fig. 8b). Later on, the climate changed during Late Campanian times to a more humid, which favored flora and fauna immigrating from neighboring areas surrounding today’s Mongolia^60^.

It is possible that *Aepyornithomimus tugrikinensis* is a transitional form between the basal and derived ornithomimosaurs. Strong similarities in vertebrate fauna and lithology are persisted at Bayn Dzak and Tögrögiin Shiree localities of Mongolia, and a locality of the northeastern Chinese, known as Bayan Mandahu (the Wulansuhai Formation)^9,60,61^. Itterbeeck *et al*.^62^ restudied the stratigraphy and sedimentology of the Iren Dabasu Formation at Iren Dabasu, which hosts one of the derived ornithomimosaurs, *Archaeornithomimus*^46^. Although this formation was originally considered as Cenomanian-Turonian^61^, Itterbeeck *et al*.^62^ concluded that the Iren Dabasu Formation should probably be assigned as late Campanian-early Maastrichtian in age which means it is equivalent to the Nemegt Formation based on the micro-fauna^62^. This line of evidence suggests that *Aepyornithomimus tugrikinensis* could be stratigraphically the oldest known ornithomimid occurrence in the Late Cretaceous of Asia, and the easternmost occurrence of ornithomimid dinosaurs from the Campanian in the northern hemisphere. Nevertheless, *Aepyornithomimus tugrikinensis* is replaced a missing gap of Asian ornithomimosaur evolution from the Campanian Djadokhta Formation, as well as the new taxon increased vertebrate fauna of Tögrögiin Shiree locality.

## Material and Methods

### Specimen and Preparation

MPC-D 100/130 was not *in situ* in the field when it was first discovered (Supplementary Fig. S2). The main preparation work was done in the vertebrate preparation laboratory at Institute of Paleontology and Geology of Mongolia by using hand tools and Paraloid B72 and acetone. The solution used for this specimen was prepared as approximately 20 g of paraloid granules in 100 ml of acetone. During and after preparation, this solution was applied two or three times because of preservational condition of the specimen was very fragile.

### Measurements

Original elements were measured in millimeters using digital calipers and a measuring tape (Table 1). Some data was collected from the literature when there was no chance for the first author to observe the original specimens directly. The photographs of specimens that are described in this study were taken using a Canon Eos Kiss X50 (4272 × 2848 pixel ratios, F-stop-f/8, exposure time-1.125 sec., ISO-800, focal length-55mm, and no flash) and a Nikon D80 (2592 × 3872 pixel ratios, F-stop-f/2.8) mounted on a tripod. All figures, sketches, and tables are created in Adobe Photoshop CS6, Adobe Illustrator CS6, MS Word 2010 and MS Excel 2010 programs. Analytical part of this study was conducted by JMP.12 statistical software (SAS Institute Inc.), and online free statistical computing the R software (version 3.3.3)^63^.

### Phylogenetic analyses

*Aepyornithomimus tugrikinensis* characters were added to a recently published data set^4^ on Ornithomimosauria. *Archaeornithomimus* was also coded. The data matrix was assembled in a NEXUS file, and is composed of 568 cranial and postcranial characters that are drawn from the recently published literature and from personal observations for 99 valid coelurosaurian taxa. Based on the preserved skeleton, *Aepyornithomimus tugrikinensis* could be scored for thirty characters (5.28%) of the 568 characters (Supplementary Data S1) and incorporated into the character-taxon matrix dataset after modified Choiniere *et al*.^45^ and Lee *et al*.^4^ *Allosaurus* was the outgroup taxon. All characters are considered unordered and were not weighted. Data matrix was analyzed using the software package TNT v. 1.1^64^. Most-parsimonious trees were obtained using following heuristic search parameters: the maximum number of the trees held in memory was increased to the maximum possible 10,000 trees; Driven Search, finding minimum length 1 times, and adjusting in the New Technology search method, was settled to using Sectorial Search, Ratchet, Drift, and Tree Fusing with default settings; followed by an additional round of tree bisection reconnection (TBR) of branch-swapping on MPTs. The analysis produced four most parsimonious trees, each with tree length of 2932 steps, a Consistency Index of 0.229, and a Retention Index of 0.591.

### Nomenclatural acts

The electronic edition of this article conforms to the requirements of the amended International Code of Zoological Nomenclature, and hence the new names contained herein are available under that Code from the electronic edition of this article. This published work and the nomenclatural acts it contains have been registered in ZooBank, the online registration system for the ICZN. The ZooBank LSIDs (Life Science Identifiers) can be resolved and the associated information viewed through any standard web browser by appending the LSID to the prefix “http://zoobank.org/”. The LSID for this publication is:

urn:lsid:zoobank.org:pub:C724A770-13E3-4076-8797-75D11D630134”.

## Electronic supplementary material

Supplementary Information
