# Author Correction: First Ornithomimid (Theropoda, Ornithomimosauria) from the Upper Cretaceous Djadokhta Formation of Tögrögiin Shiree, Mongolia

**DOI:** 10.1038/s41598-018-23583-0

**Published:** 2018-04-11

**Authors:** Tsogtbaatar Chinzorig, Yoshitsugu Kobayashi, Khishigjav Tsogtbaatar, Philip J. Currie, Mahito Watabe, Rinchen Barsbold

**Affiliations:** 1 Department of Natural History Science, Graduate School of Science, Hokkaido University, Sapporo, 060-0810 Japan; 2 Hokkaido University Museum, Hokkaido University, Sapporo, 060-0810 Japan; 3 Division of Vertebrate Paleontology, Institute of Paleontology and Geology, Mongolian Academy of Sciences, Ulaanbaatar, 15160 Mongolia; 4 Biological Sciences, University of Alberta, Edmonton, T6G 2E9 Canada; 5 School of International Liberal Studies, Waseda University, Tokyo, 169-8050 Japan

Correction to: *Scientific Reports* 10.1038/s41598-017-05272-6, published online 19 July 2017

The original version of this Article contained errors in the spelling of the authors Tsogtbaatar Chinzorig, Khishigjav Tsogtbaatar and Rinchen Barsbold, which were incorrectly given as Chinzorig Tsogtbaatar, Tsogtbaatar Khishigjav and Barsbold Rinchen respectively.

These errors have now been corrected in the PDF and HTML versions of the Article.

