# Author Correction: First Ornithomimid (Theropoda, Ornithomimosauria) from the Upper Cretaceous Djadokhta Formation of Tögrögiin Shiree, Mongolia

**DOI:** 10.1038/s41598-020-58481-x

**Published:** 2020-01-27

**Authors:** Tsogtbaatar Chinzorig, Yoshitsugu Kobayashi, Khishigjav Tsogtbaatar, Philip J. Currie, Mahito Watabe, Rinchen Barsbold

**Affiliations:** 1 Department of Natural History Science, Graduate School of Science, Hokkaido University, Sapporo, 060-0810 Japan; 2 Hokkaido University Museum, Hokkaido University, Sapporo, 060-0810 Japan; 3 Division of Vertebrate Paleontology, Institute of Paleontology and Geology, Mongolian Academy of Sciences, Ulaanbaatar, 15160 Mongolia; 4 Biological Sciences, University of Alberta, Edmonton, T6G 2E9 Canada; 5 School of International Liberal Studies, Waseda University, Tokyo, 169-8050 Japan

Correction to: *Scientific Reports* 10.1038/s41598-017-05272-6, published online 19 July 2017

The original version of this article did not meet the requirement of the amendment of Article 8.5.3 of the International Code of Zoological Nomenclature^1^. To fully meet the amended provisions of the Code, the following Nomenclatural Acts have been inserted at the end of the Materials and Methods section:

**“Nomenclatural Acts**.

The electronic edition of this article conforms to the requirements of the amended International Code of Zoological Nomenclature, and hence the new names contained herein are available under that Code from the electronic edition of this article. This published work and the nomenclatural acts it contains have been registered in ZooBank, the online registration system for the ICZN. The ZooBank LSIDs (Life Science Identifiers) can be resolved and the associated information viewed through any standard web browser by appending the LSID to the prefix “http://zoobank.org/”. The LSID for this publication is:

urn:lsid:zoobank.org:pub:C724A770-13E3-4076-8797-75D11D630134”
