# Supplementary material for: First Ornithomimid (Theropoda, Ornithomimosauria) from the Upper Cretaceous Djadokhta Formation of Tögrögiin Shiree, Mongolia
Source: Sci Rep. 2017 Jul 19;7:5835. doi: 10.1038/s41598-017-05272-6 (PMC5517598; doi:10.1038/s41598-017-05272-6)
Supplement: Supplementary file 1 — Supplementary Information [file 41598_2017_5272_MOESM1_ESM.pdf]

First Ornithomimid (Theropoda, Ornithomimosauria) from the Upper Cretaceous Djadokhta Formation of Tögrögiin Shiree, Mongolia

Tsogtbaatar Chinzorig[1, 3] *,

Yoshitsugu Kobayashi[2],

Khishigjav Tsogtbaatar[3],

Philip J. Currie[4],

Mahito Watabe[5],

Rinchen Barsbold[3],

[1] Department of Natural History Science, Graduate School of Science, Hokkaido University, Sapporo, 060-0810, Japan
[2] Hokkaido University Museum, Hokkaido University, Sapporo, 060-0810, Japan
[3] Division of Vertebrate Paleontology, Institute of Paleontology and Geology, Mongolian Academy of Sciences, Ulaanbaatar, 15160, Mongolia
[4] Biological Sciences, University of Alberta, Edmonton, T6G 2E9, Canada
[5] School of International Liberal Studies, Waseda University, Tokyo, 169-8050, Japan

* chinzorig.tsogtbaatar@gmail.com

April 25, 2017

Supplementary Table S1. The ratios of the fourth metatarsal length to the third metatarsal length and the second metatarsal length to the third metatarsal length. *Abbreviations:* (Mt II), the second metatarsal, (Mt III), the third metatarsal, and (Mt IV), the fourth metatarsal, (AM), Albany Museum, (AMNH), American Museum of Natural History, (MPC-D), Institute of Paleontology and Geology of Mongolian Academy of Sciences (=Mongolian Paleontological Center), (ROM), Royal Ontario Museum, and (UALVP), University of Alberta.

**Group**	**Taxa**	**Specimen #**	**Mt II Length**	**Mt III Length**	**Mt IV Length**	**Average of same taxa**	**Mt II length proportion**	**Mt III length proportion**	**Mt IV length proportion**
Ornithomimids	*Aepyornithomimus tugrikinensis*	MPC-D 100/130	201	211	207	0.981042654	0.324	0.340	0.334
Ornithomimids	*Anserimimus planinychus*	MPC-D 100/300	270	300	268	0.893333333	0.322	0.357	0.319
Ornithomimids	*Archaeornithomimus asiaticus*	AMNH 6565	258	282	262	0.929078014	0.321	0.351	0.326
Deinocheirids	*Deinocheirus mirificus*	MPC-D 100/127	497	600	553	0.921666667	0.301	0.363	0.335
Ornithomimids	*Dromiceiomimus brevitertius*	ROM 797	253	298	273	0.916107383	0.307	0.361	0.331
Ornithomimids	*Dromiceiomimus*	ROM 852	325	370	340	0.918918919	0.314	0.357	0.328
Ornithomimids	*Gallimimus bullatus*	MPC-D 100/10	144	157	148	0.938194444	0.320	0.350	0.329
Ornithomimids	*Gallimimus bullatus*	MPC-D 100/11	480	530	500				
Ornithomimids	*Gallimimus bullatus*	UALVP cast from Warsaw	435	470	440				
Ornithomimids	*Gallimimus bullatus*	MPC-D 100/52	256	283	263				
Ornithomimids	Ornithomimidae indet.	MPC-D 100/121	273	305	283	0.927868852	0.317	0.354	0.328
Ornithomimids	Ornithomimidae indet.	MPC-D 100/138	458	500	467	0.934	0.321	0.350	0.327
Deinocheirids	*Garudimimus brevipes*	MPC-D 100/13	195	229	212	0.925764192	0.306	0.360	0.333
Basal ornithomimosaurs	*Harpymimus okladnikovi*	MPC-D 100/29	292	310	304	0.980645161	0.322	0.342	0.335
Basal ornithomimosaurs	*Nqwebasaurus thwazi*	AM 6040	118	125	124	0.992	0.321	0.340	0.337
Ornithomimids	*Ornithomimus edmontonicus*	ROM 851	265	310	295	0.951612903	0.304	0.356	0.339
Ornithomimids	*Rativates evadens*	ROM 1790	277	300	285	0.95	0.321	0.348	0.330
Ornithomimids	*Sinornithomimus dongi*	Alashan#3	100	111	105	0.945945946	0.316	0.351	0.332
Ornithomimids	*Struthiomimus altus*	AMNH 5257	342	370	352	0.925675676	0.319	0.353	0.326
Ornithomimids	*Struthiomimus altus*	AMNH 5339	328	370	333				

Supplementary Table S2. The ratios of the length of the third metatarsal and the distal end to the medial expansion of the third metatarsals (in, mm). (Mt III), the third metatarsal length, (DE-ME), the length from the distal end to the medial expansion of the third metatarsal, and (MPC-D), Institute of Paleontology and Geology of Mongolian Academy of Sciences (=Mongolian Paleontological Center).

Taxa	Mt III	DE-ME	Ratio (%)
Harpymimus okladnikovi (MPC-D 100/29)	310	135	0.436
Deinocheirus mirificus (MPC-D 100/127)	600	240	0.4
Garudimimus brevipes (MPC-D 100/13)	229	81	0.354
Ornithomimidae indet. (MPC-D 100/14)	271	78	0.288
***Aepyornithomimus tugrikinensis* gen. et sp. nov. (MPC-D 100/130)**	211	34	0.161

Supplementary Figure S1. Possible dispersal patterns of derived ornithomimosaurs between Asia and North America. Topologies of four most parsimonious trees, including a position of *Aepyornithomimus tugrikinensis* gen. et. sp. nov. with ornithomimosaurs. *Explanation:* (384(0), 448(1)… 271(0)), the character numbers with changing character codes in every stages.

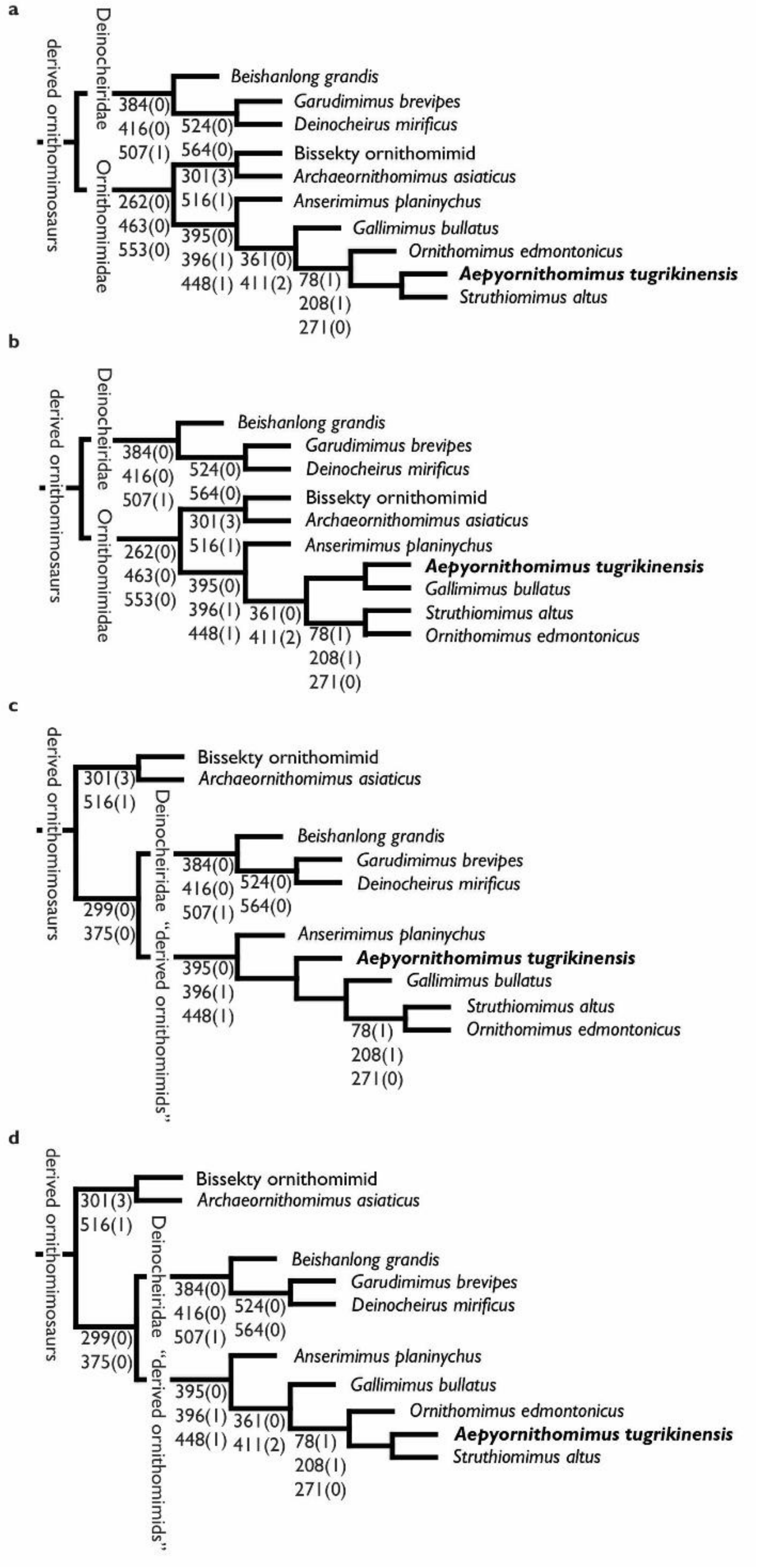

Supplementary Figure S2. Field view of *Aepyornithomimus tugrikinensis*. *Abbreviations:* (Mt IV), the fourth metatarsal, (III-1 to III-4 and IV-1, IV-3 to IV-5), phalanges of the third and the fourth digits, and (?), unrecognized parts of the specimen.

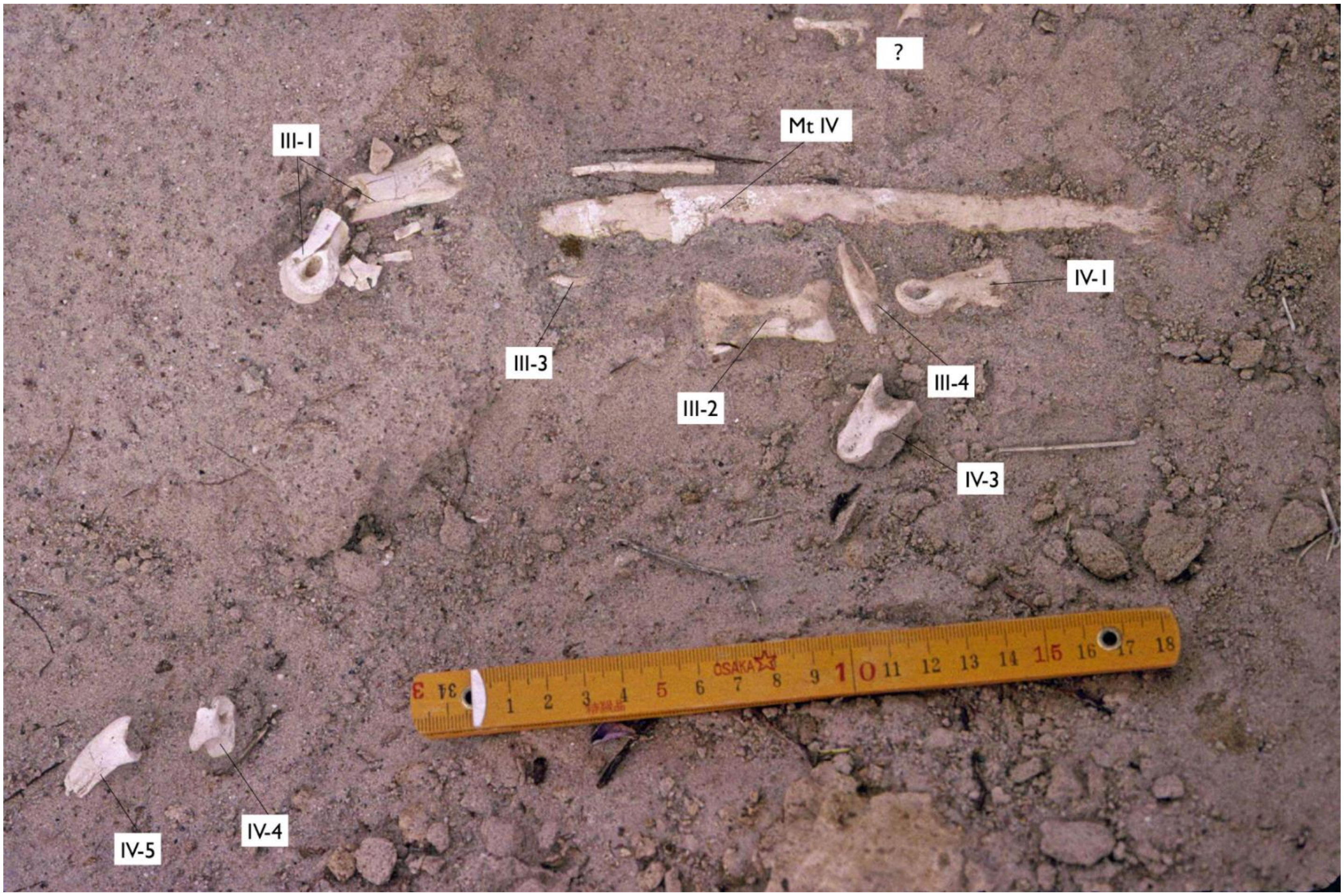

Supplementary Data S1. List of characters coded for *Aepyornithomimus tugrikinensis* and followed by modified character-taxon list used in this study.

Characters coded for *Aepyornithomimus tugrikinensis*

530. Condyles indistinct or poorly separated. The condyles are separated by a prominent vertical tendinous groove on the anterior surface (530(1)) in Alvarezsauroidea and Avialae. **[0]**
531. Astragalus and calcaneum: separate from tibia. Among Ornithomimosauria, these tarsals fuse to each other and to the tibia late in ontogeny (531(1)) only in *Deinocheirus mirificus*. **[0]**
532. Fibular facet on astragalus large and facing partially proximally. This facet is either reduced and faces laterally or is absent (532(1)) in Oviraptorosauria, Troodontidae, and Alvarezsauroidea. **[0]**
536. Fossa on anterior surface of mesial base of ascending process of astragalus, sometimes bearing accessory fenestrations is presented. **[1]**
537. Ascending process of astragalus and astragalar body: confluent or only slightly offset from astragalar body. **[0]**
538. Astragalar condyles significantly expanded proximally on anterior side of tibia and face anterodistally. A common feature for Coelurosauria and outgroup taxa. **[1]**
539. Horizontal groove across astragalar condyles anteriorly absent. Reversal to this plesiomorphic state is a synapomorphy for the clade Ornithomimosauria + Maniraptora. **[0]**
540. Calcaneum: well-developed facet for tibia present. **[1]**
541. Distal tarsals separate, not fused to metatarsals. They are fused to the metatarsals (541(1)) in Avialae and a few other taxa. **[0]**
542. Metatarsals co-ossification: not co-ossified. The metatarsals are co-ossified (542(1)) in *Avimimus portentosus* and Avialae. **[0]**
543. Shafts of Mt II and Mt IV: closely appressed throughout most of metatarsus, adjacent surfaces flattened for contact. A common condition for Coelurosauria and outgroup taxa, reversed in Therizinosauria. **[1]**
545. Mt I is absent. **[1]**
549. Mt II proximal end of flexor surface: large quadrangular flange present. A synapomorphy for Ornithomimosauria more derived than *Nqwebasaurus thwazi*. **[1]**
550. Distal end of MT II smooth, not ginglymoid. A ginglymoid distal end of Mt II (550(1)) is characteristic for Dromaeosauridae. **[0]**
551. Tuber along extensor surface of Mt II absent. This tuber is present (551(1)) in some avialans. **[0]**
552. Posteromedial margin of Mt II diaphysis: well-developed flange absent or area rugose. This flange is present (552(1)) in Oviraptorosauria, Troodontidae, Alvarezsauroidea, and Avialae. **[0]**
553. Distal end of Mt III: smooth, not ginglymoid. A ginglymoid distal end of Mt III (553(1)) is characteristic for Deinonychosauria and Avialae. **[0]**
554. Mt III pinched between Mt II and Mt IV and not visible in anterior view proximally. A synapomorphy for Ornithomimidae. **[1]**
556. Medial side of anterior surface of distal end of Mt III unexpanded. **[0]**
557. Mt III shape of shaft in cross section wedge-shaped, plantar surface pinched. This derived character-state is found in all Ornithomimosauria except *Harpymimus*, *Garudimimus*, and *Deinocheirus*. **[1]**
558. Shaft of Mt IV round or thicker dorsoventrally than wide in cross section. The derived character-state is present in Dromaeosauridae and a few other taxa. **[0]**
559. Length of Mt IV markedly longer than Mt II. This character is present some Ornithomimosauria except *Anserimimus planinychus*. **[1]**
560. Posterolateral margin of Mt IV diaphysis: well-developed flange absent or area rugose. This flange is present (560(1)) in Deinonychosauria. **[0]**
562. Pedal digit IV is significantly shorter than digit-3 and subequal in length to digit II, foot is symmetrical. **[0]**
563. Extensor ligament pits on dorsal surface of phalanges of pedal digit IV shallow, extensor ridges not sharp. This plesiomorphic character-state is characteristic for all Ornithomimosauria except *Nqwebasaurus thwazi*. **[0]**

564. Pedal phalanges of digit IV. Anteroposteriorly long, proximal and distal articular surfaces well-separated. **[1]**
565. Shape of ventral surface of pedal unguals. Ventrally concave in lateral view. Except *Garudimimus*, *Deinocheirus*, and *Qiupalong*, which have pedal unguals that are ventrally concave in lateral view, other ornithomimosaurs are presented a straight in lateral view (565(1)). **[0]**
566. Ungual and penultimate phalanx of pedal digit II is similar to those of digit III. **[0]**
567. Ventral surface of pedal unguals with a pronounced flexor fossa on ventral surface of proximal end. A synapomorphy for Ornithomimosauria, reversed in *Qiupalong* and *O. edmontonicus*. **[1]**
568. Form of flexor fossa on pedal unguals: without development of flexor tubercle. In ornithomimosaurs there is often presented low longitudinal ridge in the flexor fossa instead of the flexor tubercle. The flexor tubercle or ridge in the flexor fossa (568(0)) is distinct in Alvarezsauridae and Dromaeosauridae. **[0]**

Six characters are assigned as autapomorphic characters of *Aepyornithomimus tugrikinensis*, as follow, a presence of notch on medial edge of the ascending process of the astragalus **[ch.535 (1)]**, a presence of fossa on anterior surface of medial base of ascending process of astragalus **[ch.536(1)]**, shape of proximal end of Mt III **[ch.555]**, markedly longer Mt II than Mt IV **[ch559(1)]**, rounded and shallow extensor ridges present at depressions on dorsal surfaces of phalanges of digit IV **[ch.563(0)]**, and anteroposteriorly long, well-separated proximal and distal articular surfaces of digit IV phalanges **[ch.564(1)]**.

Data matrix

#NEXUS
begin data;
dimensions ntax=99 nchar=568;
format missing=? symbols="0~6";
matrix

Acrocanthosaurus_atokensis ???010100?0??101?0??10?0?10??0?1?0110001001000?0?0?0112011??0?000010
10??1110???11?0??0??0?01101111?00??10110??01001?1120?0??1?0??0?????01?0?0????1??0101?0?10000??
??10??????????0?????200??0000??10???0?10111???0??0?0??101??000021001?00?0?0??1?0???1000?1??1?11
1110??001?11??0?10??2?1??01????10?1?00??01?0?1?????????0?02??????10?1?0????1?010?????????0???1?1
01?10?0?100000??10???02??00?101??0?0??01111?0?10100220002??100?0???00???10???????????????10????
??????101010??0?0{01}0??0????00?11?10?00???01012?1001?011?0??00100?101?????0?0?????0??0??100??
??001?000?00??0?1010??2?01000?
Afrovenator_abakensis ?????0???????1?101001??0??11010100110?00000?1???????0?201100??0?0010100111
00??00??????????00010?01??0????010000001?010????????0?0??
???0200010000???????0100?0110110?10?1?0001?1?1
1???0100?{01}?1010?????1?00?????0????????????0???0??0?0?0?100???????11?00?????????????????????????
?0??0?01??????00?????????1???011?1?0?????12100?2???01000000100100??0????00?00010000100?000000
?0?0?01???0001000100110100000??0?0?11000100010001000????0?0??????001????00?0000100?111??1?0?110
????01?????2???0???
Albertosaurus_sarcophagus ??1????0000011??0????10?1???0??0??0100000101110?11??00201100110001111
1011201??000?12000001011110011201010l?001010100103000001000000000001?00010?1??1000?0011000
0100?10??00???0000?001??00??0?00?00?1000010111?011100???010??00?10?00??0?0000000?0001010???01
1?0???????0000011?00????0?0?0????????1??0000?00?00410?00???10??0??00??0??0?00?0????0??0000??0100
0010???00?000011?001???????00??0?00??0100000010?0???????320?0????0000????????10101?100000000?
20???1000000?010??0001???00010000000102000101?1101100110000100100000001?1110011001?1000100?
2000110?001?0011000001111000?0?1000?
Allosaurus_fragilis ??0010{01}01001010?0000?0000?1000010?110?0100100001?1?011?1?000000000101???
{01}100???1110100000?01000?010?0???01?01000101011 20?00010??000100101?00?00?10?0010100110010
0?1?10??0?0??000000010000??0000?00?1000011111?0001001010102000000210010000000000000010100000

111010?1000000101100001001101000100001000000100100300000000000000200000010010000???1?0100???
100??0001101010000001000000001000000000000000101000011110001010121000211100000000010010100
0100010000010000100000000101000?1010000100000011010000?000101100011001100100010001011000
0?0001000000011000111 0?1?00110000001010?02001000?
Alvarezsaurus_calvoi ??
??
??0????00?0?0?0?001210000?1?
??????????????????????3020????????12100?0?1002????????????1??????????000?01?00?000??????????????????
??1?011?1101000?10020??01100210020011 01?00??????????????????????
??????????????00?11?1??001??????????????????????10????????1011??11111001??????00?00?01110??10000?
Alxasaurus_elesitaiensis ???????????????????????????????????????0???????????????????????????????????????
??
?1?00110101??10?101?0????????????????????????????00110?10211000101 0?0??????????????????0?????????
0?00?010010011??00001001004?1??0??????????01??00?2?01??????0000?????????0???????0???0100010011
101101????00??0?100010000000?11101220?02???00100100???110100?110101?0???????0???000?2???????1
?????011110?1?1?220?0????????111?00???0??0?10????????????????1??????????????????00?010??00?00???0?
0?001?0??
Anserimimus_planinychus ???
??0??????????????????????????????0??????????????????
??1???
??????????????0???0????????111100???10000?12????????2??????
?????????10000?1010011010?00221??111110012210????10100?100?11?0???????1010000?010????00???00?
1000100010??000??001?1????00?010110???0??1?
??
Apsaravis_ukhaana ???????0??
????????????????0??????????????????1??0??
001010011100?0?0??2???????????????????110?1?001?12110??1?11?00?
?0?????????????????0???501210?0?210?10?0200???2?0110????????11????103000?11100110101100?0001101
1100010100001111100 01???1?11?1???????????????????11000?01010??0???1?1?1?000?10????312??1???1?00
000?00000?1?22000??11?11112?2001?????1100????????????11????????11?????110?111?00??000110?11010?
001000?
Archaeopteryx_lithographica
110000101001000101 0?1?00?00?000100010001???0000010001100111100021?0?0??1100111110?11010000?
?0?0?000000?010?00?000???0?210??12?010?0?0??????????0?0?01110001???0???????1001011?00??10010000
??0000?00?0?0?00?0?1?0?00?0??0010100010020 01?212010000?00??10?0??1???0?0???????10??????0??????
?0????????????00???0??04????0???20?01??01??1??2?00?0????1?1110??101?2011?0110001111110020??1?00
010????000?011?10?00?0?0?1???1?22??0??2??0000000???00?010???1001?1??0????0?01100?2?1????11???0
011210000010?2101001??????11?0110?0??0?00??0???????0010?????00?0002000110?11110011?00?00?0?010
?0??010?
Archaeornithomimus_asiaticus ??
??
??202?1000?011
2110000??200000010??00?000110301010?0??0000200?0000?00??0??????????????????111110??1??000?120?
?00?120000100000000???????1010101011100?2?0?1?????01??1??100??1???1000110??1000001000????0100
?0?0?1??00010001?00101?000??1?10110001100?000100010001??111001??????????0????????1??001?????100
001011000??001???
Avimimus_portentosus ???????????1?1???11??1?????????????????
??????0?000?????0??01??????0?00?000???11?????????????1?1?0?1?10?00?01??120?0?0??0?00??110????????
?0??00????11?????????11??10?0?0?00???????111???????????????2??2???????????????110?1110100120 00110?
112110100101000111100000000110601010?0?2?0000???00???????????????????????????2001001100001001 1

0010010100010010000001?111????0???????0??2???????????????0100??1???10000111010100001111?00?01
00100011?00011110230010?0000?01101121011010000010100000100110011?1110001011200011011100?
11000102?110102010100?
Beipiaosaurus_inexpectus ??
??0??????????????????0
???1?00???1?1??10?101??100?????????????11??????022000110013201000101?0?????????????????????????1?
??0???????0?????????????????0????????????????????????????0????????????00??1001001?10000?0100???????
??10????????0????00100?????????0?22?????1?00?0000?01011110011??????1???????????????????????0?????
??????????0???????1??????00???0?0?0??0????0?????010????????10?0??0??????001???11??????????????????
0?
Beishanlong_grandis ??
??
??1?11???????
??1??????????1111101?01000?0020??000010000000?
???000????????????????????????????000002?0???1????
???????10?0??0002100110?10001010???1100010?0?1010100?1000110???1?001?????00??10????001011
Bissekty_ornithomimid ???
?1??0010010???
???10?102022100?01121100
00102000100100000000011?30?01?000?0000000?0100200??0?????????????????1111101?11?000??200?0001
????010000????????????101?101111100??10???????01??1?010???????100?110???0000?100?????????????????
?????????????????????010110001100100011000000101111?0?1?100??0010002000110?001?????100001?110?
0??001?11
Buitreraptor_gonzalezorum ?????????0000??1?10???0?1???0??????????1???????0???????????00????0???????
???????????1?10?010??0?001?0????0???0?10?001??????????????????????????????????????0????????????????
?????????1?0?0??0????01010????????????????????????????0??01?0020???00?00??00?0??110?010???2012101
10??12??010???????0??1????0???1?1??01??0???????1??01??10?2?01??????1??2????10??2?1????11??101110
01??????????0??1??????1?110???????????????22????????????????0100??????????01?10?0????111?111?1????0
10????20112122??0112?0?1001???????1??1???????????????????????1????????11?20001????01?0011?10??1?0
1001?0???1??
Byronosaurus_jaffeei ??0000001100000100001?100110?000001110001????00001000??0000???0????110??111
01110?0??0???000?11?0?1???0??011?100111????010???110010
10111?????0?000?00000?0110?0?010???????????????101?001002201?002000120010??10?0??10???????????2?
?0??????210?1????????????1??1?????0????????????????????0???2??
???1?
???????????????????????????????1????????????0?0?01??11????????????0???
?1??
Carnotaurus_sastrei ??10000000001000???0??00?010000100010001???1?1001110??200000000?000?1010100
0???10?11000000001?11011001010000010000??00????0????1000??2??0?????0??????00?1001??0?0?????????
???????1?101100??00?01000?100?01000??0?11101000101000??01000000?0?0000000???0?00001010101101?1
0?00??11102?10100?00100?10?10000111?110040?11?0??????00????0?????0000???0??????00??0?0000??010
01010?221?0110?200000?000111000???????????????????????????????????????11000?0000??????00?00??00??0?
00000100????1000000100?000?0?00000???000?10000?0111011??011?00?110?0???????????????????????????
????????????????????????
Caudipteryxz_houi
100000??0?111?0?0000????????0?0?0?11??????????00???01?????000?01?0??????1?0101?10??10?000010??0
?00??0???00????000??0??????????????1?????????????????????????????????0???????????????????0?201?0011011111
00?110?1??0???0?0?????0?10???1101??????12??2????01???0????????0?0?????1??00?????????0?{01}0???00?
???????????????04?1??0???2100??11????0??00?0???1?011?00?110??00????1?001?0?0????1??10?0??0000?0?

?0001?110?0000?0111110?22??021?100?00000100101001???0?1?10?0?000????00???????01???2011110?
?00102?0?0??100?111111?0?????10??00????????011???10????01200011?1001??0????01?1?0???1?0??000?
Ceratosaurus_nasicornis ??101000100011 00???00?00?0100000001000010001111010101101?1000000001110
?11100??0010010?000000010?011101010010100010?00?0?00?0??1000?02??01???000??0???1?1???10?00???
0????????????1?????000????0???0?10?001000?0??????100?100??000?11000?0000??0?0?0??00?0000111?11
011000000011??0?00101?00100????100000110110 04001?0?1?000?01000?0?0100001 0??10000????????000
10?01001000??????????????10001100101????0100?100010101110??1??????2????1100??0000????1?000000
100100000000?000101000100100000010?00000?0?1100000000001101100011101111 0?0?0000?1000???00
1000011?????????????00?0???????0?0?
Chirostenotes_pergracilis ??1????????1?00??????1101??
??1?0100??01?1??0???120?011??0?00?1?110??????1
????10?0?01111111100?100?10?0?0??00??0?????????????1??????????2??????????????????110??211??0????
?12???1??????010?1??1?????????5210????0??1?0??????????????0?????????????????20?100???0?10?????????
????????????????????????????0???????2200???????10000??1000?100010000??1??010000?000000?12??00???
??20111102300112 1000?01????0?0??????0?0?11??0?0??????00??????????00?????1???0?1??0??00?01?010?
0?0??00??
Citipati_osmolskae ??100000001010 0101?0?110100?00020111000????0000100101?0010100001000?1101100
101?10?11110000100?0?001200?000?000000??010?01101111 1101?0??01?01010?010112000000?0?001??11
111110?0000021?0201101111100?110?10?01010001100?0111??????1??????????2??????????????10??110???
???210010??1????1?0?1???????1??1000??0?1?52???0???10?????1???0??2?01?1????0??0001111 11200?????0
0?110010?1?????1?00?0???000??0?1?10?00???0?????0?22??0????0010000?????0?100????001?1???????0?01
000?110??0110???20?11102300102?000?01??????2110010?0?10?????0????????????????0000??000????001?
?0???00?00?0?0?0?0??000?
Coelophysis_bauri ??000011??0??000???????0?00?0101001?1?10???0?00001001????0000000000?0??11100?
?00100??0??0?000?0?01?00??0??0?????0???????0?????0000?0???01?????0????1???0?0?1000?????????????
???0?001?000?0?0011???10?100??000??????????1010?010?020000000?????000??00?1??0100?11000?02??00
10?0?10000{01}0002001000????0??00?0?03?00???100000020?000100?01?0??????00?????????0????0011?00
?0?1?0?1000010??0100????000??1?0000001?00?0?0?100??111100?0???001000?0000????100000001101???0
00??0?00???0001101100????10?1?0?0???1100?0??0?0000000?00101?0?110???0??0?001????001000000??0?
??10???0?0?0????1001000?
Compsognathus_longipes ??00?011000????1010?1?00??1101000???0000???000000000??00001000000?0?0??
111?0????1?1101??1?000?0?001000?0?0??????0???????????????0???????1???????????????010??0?????????
?????????0011000??0?00?00?0?000100?0?000??1210?10100010021011001000000000 0100?0??0??1010?1?
0?0?00010??0?1?0?10?0200????????100010?103????0??0000?0?000?01000010000?1?1000???1????00010?1
11?00000?0??1???1?0??01???000000????0?0101??0?10?1?22??12??10000011001??0?1000???0???0??0?0???
????00?01111???0010000100010 0010100?0??1??????000??1????1???00?0??????00?0??????0?0001000?10?0
01?0011000?00?0?0?02001000?
Conchoraptor_gracilis ???0????0011100?0?00??1?????0?020??????????????01????1????110??010?0????????1
01?10??10?00001???0??01??????0?000000??0??????????1??1?????????????0?????0?2???1?????00???110?????
???0??21?0?01101??1100?110?1??0?0?000??0???111??????1??????????2?????????????????????????????0???
???????1?????????????????01??0????11??0????????1????????????????????????0??????1?12?010???0??0100?0?1??
?????00?0???0?????????????????0?????0????0?????00100100????0?100?11?001?1????????0?10000?110??00?1
???20???1????010??000?0????????11?001??0?10?????0????????????????????0???????????01??0???00?00?0???0
?0??0???
Confuciusornis_sanctus
11?000?01?000000????0?01?0??00110011000???00000?0?01?11?111001?1?0?0???1?0????10???00?00?10?
?0?000?0????0?00?000??1??????????0??0????????????????????1?????0??????????????????????0?10??000??001
1000?100?0100?1?000??0???011???????1???????????2??????????????????0????????????????????0????0??0???
?0??100???1??05????0???20?01??1??0?????010110??????11111010?0?????1?0?1111110020??110111 0001?1

000011?11?00?0?0?????1?22??0?????000000?1?000?010????0?1?1???????0???100?30?????1????0010?1????
?1?211011?11?????2?20?1?????0??0??1??????00???????01??1??2110???111100??001?10?01??0?0??000?
Cryolophosaurus_ellioti ???????????????????????????????1??1?1??000??0??0????0?2?????0?00001011??110
0??001000??????00000?01?00??10?10??00????10?0?0???????0?????????????????10101?????000??????????0
?????0????????????????????????0?0110??0????????????????0{12}??0?0000??????????????????????10?1{01}???
?00?????0???0??????00????10010??1?10?0???????00???0????????00?0?0????0?00????????????????????????
??????0????????10000???00??????????????0?0??1??10?????00????
????00????????00????10??????01001?010??011?0??0000??????????0??????????001100000?????????????????
????????????
Deinocheirus_mirificus ??00000021001001001?0101110?0013011?000????0000000201101001100100?0?0??
1010110011200010001?000?0?000000?0010010010010011????????010?0?0???1?1?????????0000001??000????
??????????????10??001101110?00?000000?1?1??000?0???011??????1???????????2??????????????????010?10
201210000?112111120001110100100010010110041201002 11?00020000000000000110?10100?????111??2001
00111000100120010001200000000000000011?00010101011111002210021110100000011011100011 0001100
?000000100000010101000010000000?0010????10000?102000100011001000100010001011011?010200001
010200001000010?????00000010000?0000010
Deinonychus_antirrhopus ??000000200001?111101000?00?000000010001000000010000110??1000001010?0
??1???100?110??1???0?10??0?00??0???1001000?0??11031?110100?0???????????1????????00??0????0?0????
??????10??????0011?00??000??00?0?000101?1001???11010101100000 2100?001000000?000100?0001111?1
0?00101200011?010110020100000000100101110 1100?????0?????00120010010?1001?1????1?12??????1?21
110??110?101010?1?0???100010001000?00?1110000111010111112200021 2?0010000001?00?1??110000 1?1
??0?0000?111001210??0101???00111122200102101 0?0101011111111100001000001011111?00101??10?000
0020001101001100 11?10?1000111010010 10?
Dilong_paradoxus
0?100020000110?101001100?10?00000011100???01021010001201100110000 0?11011100??0010001100100
0000?001200?0011?01010??011??0?????00000100101?00000??????1?1??1??0001??0?????????????0?0?11000
?00000?00?0?0000?11111111101010101000010200000000000001000001 00?0???????10?1?100000010??0010?
0?0101?????????????1????0??????????????0?0???????2?000?0??1??????????????0??????1?1000?011???10001???
?????????????????????1?1?1?1110022???21????00000??1?0??1??010000??0??20000100?0?0??11?????????????
??0???????0??0????11?11?0001?00??0?10??0?001?111??010???10???0002000111??01???????0?00?0??0?2???
000?
Dilophosaurus_wetherilli ??001020?00??0?0????1?????0?010?0010011{01}0{01}1??0?0?1001?20100010000
10?0??1111???0010?0????0?00010?01?0????111????000?011????????0000?0???01???000??1?1?0?0?0?1000
0??101?????0????????1?1?1?0????0???0?10?00101?01??????????1010?000?1000000000??1?10???00?011011
0?1101?0011?0011?0??0?011000200?10?10000??1100?03000???0?0000020?000?01000?0???10?0??????????
0??100010?00?0?100?10000100?0100?0??010?01?00?0001?00100011000?1?11000?????11000????0????1?00
?0001101????100?0?00???100?00?100????10?????0???11000001000000000?0000??00110?000?0??100????0
01000000??0?0?10??0??0000?0?2001000?
Dromaeosaurus_albertensis ????????2?0??1??1?1??100????0???0?010??10100??0????011???100??0101?????
?1???????0?121?1101???????0??0?????????000??01031???0?0??000100001?00000?0?0?0011?01010000?111
0?10?1000000000011000?00000?00?0?000100111010111101010?000110?000?00000000000?0100?0?????????
??0??????????????????????????????????????0??
??1???????????????????
??
?????010?
EK_troodontid ???
?????????????????????????1????11??????????0???????0????????????????10????1????1??0?????01????????????
??????????????100???0????1????????????????????????0???????1???????0???????????????????????????????????
???

?1000010?01????1?22?0?2???0000???0??????????????????????
??0011?00?1????0???1???10?
Eoraptor_lunensis ??0000000000?000????0?00?0100100000100????000000001?01?0000000000?0??11100
??001001??0???000?0?00??0??00000000?0???10???0???0000???
???0?101?000?00?0??00?0?0?010?0??0?0???????10100000001000000000??0011?0?0?????0?0?????????0?
10?????0?00??0?00????????0??0????01???????0????0???0?00???0??0??????00?????????0?????00??00?0???0
?10?0??????????0??0????????10?01???0??0110???0100???0????00000?0??0???????0?00?100?????????0????
??00000?010?????00?1???0?????0????2??0?0???0??????????0?????????00????????????????????0??????0????
??????0???
Epidexipteryx_hui ???00???0???????0?001?00???????????????
?0???0?0?0??0?001??10????????00???0??10?2?
0?00???1?10?00?10001??001?00???????010?011100?01??2120?101?0?1??00?0??????0??????????????????12??
000???100????????00?0????04??0?0?0?2100???020?1??2??100????????10??????2?1?????0?100?101?02?????
00010?0?0??001????????0???????????22??0??????00001?010?0?200011??????????0???????0??00??????????0
?010102?????110????1??????????????????????0?????????????0?????????1??0001???11100110????0?0???0????
????
Erlikosaurus_andrewsi ??100000100110000??0?101110?00001101000????0?00???11????1000001010?0???1
100???110?100000010000?00100??0000010000?0??0???1012100001???010????00?0?1000000000???00?????
????10??000?1010?011?1011010?101000?011?0000?0100011??????0220001?0013111?001010?????????????
???1???
?10??
???0?????????0??0???????
???0????
Eustreptospondylus_oxiensis ??0??020100?1100????00000?1100??0001001000?0???????1?????????000?1?1
1011000??0011011?00?000010?010?0????010?00000?00?????????0??0?????01?00010?01010001?0111?100?
??10?00?10000??0??1?110??0????0101001?????????????0?????010000002000?000000101000100?01101110
10?11100101011?00?1000100020000001100000100?03000?0?0?{01}00002???00?00?????????????????????
???????1?00100?0200010001?000???10???0??0???01000?00
00001100000000100000010100001000010?0?01?000?010100100000000100010001000000111000000001100000000
0100011??00??????00000100000?001000?
Falcarius_utahensis ??????????????????????0????????????010??001?0???
??10?0000?0?00?00??0????????????1?10010???????????????1?0??00?00?1?01000?000??0?011?110?10011010?
?0??1?00100000??00?1010?????????????????????????0?20001{01}00101110001110?0???????1101?2121000
10?11211112010200111010010 10?001004110100 0?1000120010011000010???101111???1010?10010?1?1010
01?1001?0010001111001100?000110010010100011110220002111001000000101111001110101 01?00000011
010??111001010101000112102300102 2000?010?011010110000000100000101111100010?11??010000100011
010001001100??001000???001000?
Gallimimus_bullatus ??0???00210000000101?101110?0?000???000????00000000?11001?110?000?0?0??1???
1110112000100?00?0?0??000?0?001?010112??00???01?010?10?0?110?1?00001?010?0001001??00011??110
?0?100010??10?0??10?1010?00?0?0?01?1?1?1000?0???011??????1???????????2??????????????10??010???20
2210010??12???0?0??????00?1??0000??0?00401??0???100?0??00??0??0?01?0????0??0????????011100??11?
000002?0?0???2?0000??0000??1?0??001010?1?????0?2???0?12?00011010?1?010100?100011?0??0????1010
000?010??01001??0001000100 01010000?110?????00?1001?0????00?00??????00???00?0?0100??0001???001
01???100?01?1?010?0001010
Garudimimus_brevipes ??0000002?000001001?0101110?00000111000????0?0000000??01?0110000010?0??1
11011101120?01001?000?0?00?000?00?00?00?001?113?01?01001000?11?0???0001?01?000010?01100?11??
???????0???00????0001001?10?00?000?00?101?1000?0?1??11??????1???????????2?????????????000??1??????
???????0????2??00001010001001???0000001?04010100?0??0000???00?0??0??0???????????????????????????
??0100111000100 11100?100100100100

0101001000101020?????????????????1010110001100100011001000101110000?0100?01000200011?0010
0011100000?1101020000011
Giganotosaurus_carolinii ???0?00????????0????10????10???1?001000100??01?1?0????2???????????1010??11
10????1?0???????01101111?01??1???????1?0??10{12}??????????1???????????0???0???101????????????1???
??0?????1?????201??0?0???10???0???1?11????????????1???000200?0?10?0?0??0?0???110001??1?111110??
{01}?1?11??0?100?2?0??0?????10010??1?01?????0???0?000?01??????000???0???1000??????????0???????1?
10?0??0???10000?110?1??0?0?000?11?????1
01010??010100?0???????11?1???????01012?100??01??0?????0??10111???0?0??1?00????1????????????????
?????????????????
Gigantoraptor_erlianensis ??
??
20?0001111011100?110?1??000?00?0????????????????????????2??
???????????????????????????????????101012?0000?0??0???????????1???????????????1?0?100??110?110002?
??00?00000000?????000101000??
???????????????????111110????10001??1?????????????????????0?0?
Gorgosaurus_libratus ??1010201000011?1010011001100?000000010000???1110?11101?0011001100011111?111
10????0??121?0000001?0?00?20101011??10?0?????3?0000100000000??01?00000?????????????000???????
?????????0?00??000?00000?00?100001011110?0??0????10100001020000000000000000000?????????0?????
??0?0??????00??0??0??0000?0?????00?1????0???????????????????????????000???????????10??????????1???0?
??32?????????????????100101011100????0?12100?100?000?0
101?0001???00?10010?0010????????????????????????????00???????????0?0?10???????????????01000110???
???1????????0?0?
Guanlong_wucaii ??1010000000111110100110011110000?011100001001011000101201100010000?1101110
0??0?100010000?00000?001200?00110?1012??0??300?1???00000100001?00000?11010001000??000100010?
10010?0000?001?000?00000?010100?00?1111??1??0????10100001020{01}1001000?000000100?1?????01
0?1010100001000?1001001001010000000100100110010030001000?00100000000?0??2000000?????11?????????00
010?1?10000?0100110000100111000100000101000000101010111002100021210000???00100101010100000000
0?0210011001000011011000100000001000000000?10001001?01100011001100100000101111100?2000?1?00
10001100111100?1001100000001010002101000?
Haplocheirus_sollers ??00??0011011111010111001110000100111000?0?0000100001?01?11000000000?0??10
00111?1120100000??10000?00??00??010011000??010?001101000000021?001?00010?010?0111??00?00110???
???1?110??00?1011110?0000100100?000111010011??02100101000100120110010001101000100?0??????00
????101?00?10?1011101001101??00001??1?????10103000?0?0?{01}?00??00100?00?00000??1?01???????????1
101001110000101001101101100?00?1100001?10000001101111100220112121010100001010???????11??010
02?000001001??00011010100??01001100000110110?0?01??011010100000?1110001011110?001011010??0
00?20???1?10010????00?00?010002001000?
Harpymimus_okladnikovi ??00000021000?010?0??1011?0?000?0?11100????000000000????????0???????0??
1110111?112010?00??000?0?00?000?00100100??????????????????0???
?????????0?0010001001011000?000001010??0000000?0011??????1?????????11?0??0?????????????1010????0
??0000???02?0?0001?2??0?00????00110?110401?10???1000020000?0000001????0?0110?????????11???1?11
100?012?0?000120000100000000011?1000001010101111022100211100011110010010100100????11?0??0??001
00?00000????010????00??????????????????0101?00???0?????????????????????10????????0001000110?001
01???100000?0?000?0001011
Herrerasaurus_ischigualastensis ??000000?00??100???????0010?00000000000????0?00000001?20100000000
00?0??01100??0010000???0??00?0?00?00??00?1????01???11??000?0?0000?0????0???000???01?1?0?0?0?00
0????0????????????0???1?000?0?0011???????010?000??????????1010?000?010000000???0?000??00?0000100
?0????0010?001000?0000000000000000100?0??1??0?00000???200000000?000?0000?????????00?????????0
??0?00?1010?00110?1000010??0100?0?00001?0??010000?10000011000?11100000???0000?0?0??0????00?0
00001001????00??1010???00000?0101????00?1?1?0???010000?2000000000000000?0000????0?0??100????0
01000000??000?0?0?00?0000?000?01000?

Huaxiagnathus_orientalis ??0???000001?0?10100??00??11000???11??0????0000?1000?????110?0000?0?0???
1?0????1???1??????????0??0??0????0??????????????????????0???
?????0?001?000?00?00?00?0?0???????????????????10??0??00?0?1?00100000000?0100?0??????0???????????
11??10??0??0?0100????????10?01??10????????????0????00?0?120?1000??1?0000???100??00010?111000000
100??????????00????00?00?0100?001??0??10?1?22??12111?000000?01?00?10001010???0??0?0??02??000?1
11?????????0001000000001010000??1??????0??????????????0?????????????0???0????00?2000110?001?0011?0
??00?0???020??000?
Incisivosaurus_gauthieri ??1000000101110100000100110?000111110001???00001000011000100000?1000?10
01200001000?101000001000?00100100000000010010102011012101101?00001?0110??11?012001000??00
10?11??1100???0?0?2011?01111111000?0?0000?000?0?0110??0?101200?00?00??1?2001101?10??10???????
??
??0??????????????????0?
??
???1?????????
Limusaurus_inextricabilis ??10001010011101011?00?000?11?0?1?01?000????00000?000??01??000000000?10
012100??00120??0????00000?00?1????0000?0?020?00??????01?000???
??????????0?20??00??01?1110??0?1000??0?10?0??0????11??????1????????????????????????000??0?1101?1?
2?0000???01?0?0????????????????00?1????0?????????10000?000???020000000?0??1101000?1??0001000110
10000?10011011?00?00000?000000?????0000?00010101{123}10?00011??02???000000?000110001000?100
100100100000100???0?0000?00???000?0?00?00?1???00?010?0?0?????00011?????????????0??10??????1?????
??001?0???00??0??00???2?00000?
Linheraptor_exquisitus ???000002000?1?1101?1?00?10?00000001000????000?0100???0001000101010???11
10100?10?1?10000?100?0?00?200?00001010?0?0?10????????0000????????????????1?10101????????????????
????????0??0??000?000???00?0?0?01011????0??111???01?????02101?0?1000000??00100????1111010?1??0
0?0??11?11??1??????????????????00?????10?????????10?0???000??001000?1101???1?111????????????1???1?
???10?11???01???1?????10?????????????????????????????2?????????????1?0??????????????????????????????2??
??010????01???????????????????1????????????????????00?101??111??10???10???0??2000110??011001??10?1
0?0?10???01010?
Mahakala_omnogovae ??
???10??000???0??????????????????1001??1???????????10?0?10???????
??
??????????????????????401??0????????1??01??10?2??????????1???1???1
0??????1110?0??????????????????????????????????1000?0???0???11?1????????0?11100?????????????????????????
??????????????1?11?111?0?0???0?0????????00??????0???00??0001????01?00??010?10??1??0?0??010?
Majungasaurus_crenatissimus ??10100010001000????1000001000010001000011100?10110101120100000000
10?101?1010??010?110000?0011?1101111101100010001 0?00?0000001?1000012??01?00000?1??1?????0???
0?0?????0???????????0?10110???0?00?????10?0?10001000010010001010000000110000000000000000100?000
0111010?1?10000?011?0200010100010011 01?000000101100 30??1?0011000020000000110000000?100100???
??????000?00??0?0?0022100100120 00???????????0???????????????????11????????????????01001000001 0001
000?000100100000010?????????????0???0??????????0???????????000000??001000111011010100001111 00010
000100010??0??0???0000000?0??0?0000011
Mapusaurus_roseae ?????0?????????0????10????10?0???001000100?1?1?1?0??1?2??1???????01010????1????
1100???????011?1111??????????????1?01?10???
????201??0?????10???0???1?1???0??0101??????????0?00?0?10?0?0??0?0???110??1????11111???001?11??0?1
00?2????0?????10010001?0????1??????????0?01??????1001??????1???0????10??????????1?1??0???100?0???1
1?1??????????????????????????0??0???????????????0??????0???10000?110????????00??110????10?????????????
?????00?11?10?00???0?012?1001?011?0????10??10?110000?00?0?00???1?100011??0???????????0?1?10?????
?????
Masiakasaurus_knopfleri ???????????????0????100???0?00???0100??10000?????????????????????????????????
?????0??

????1?110?01?11100?1000?????????????0???????????0?00?0?00?000?00010100?0???????1101?10??00?0?1?
2110?0???01??00001??00010?01?0???01?0?110?0020????0??0?0???????????????????00010????????????100?
1001???110?010000
00????????????????????0100100010000100100011101011000000?????????1??1001111001?????000?0???00?
???001?0?
Megalosaurus_bucklandii ?????000100?1?000???????0?1000????1100001101?????????????????????????????
???
?????1?000??00???00???010100????0?????????????????0?0001000000?0000?010100????????0??????????????
????0?{01}?1??00?????100?????0???300010100??0??2????0??0?1????????????????????0??0??0?0??????201
1?0201??????00?0???010?10000110000000?01000?100000100??00
000????000100010?1??0??????0000010000000100010?1010101100??0???????????????????????????????00
1?00?0????????
Mei_long ??00000011011?00????0?00?12100000?11000?????00001000??01??11?0?11?0?0??12?01110?0?11
00?000??0???001100?0???????10???11??????????0???????????????????110??????0???????????????0??0?????
?00?0000??0110?0??????100????1????10?0?0?001201?0020???0???0???0?0??110000????101210110??10100
1?0?11000?00????00?00111040??1??0?1000?0001??2??0?0?0?1?0???12???1111?20110?1100110100000010?1
1100110???????0??????????????1???????2??????????0000001????????0??00??1?0011000?0110000????01001?
0101??10???0??20??10?10?01??1110110?0?11?000?????????011?0?100??0112000110?00100011???1?1??10
112101010?
Microraptor_gui
100????01000???1??????00??0?0???????????????00000???0?????1?0?0???????????1?0??????????????0??0??0?0
0????????????????????????????????0??10?0???000??00?0
?0????????01???1????10???01?02?0??0?10000???00??10???????????????????0?111??1????00??01?????????10
0?0?1?004???1????10??1?001??1??1?01?1??????120011101??0???????1??111110?11???0???110??????000?11
0000000101?0??0?22??02??1001000000100101?0?111??1?1???????0?011?1?20???010?1?0011121?2??01101
1010?1??????11?111????10???0?0??????00??????0?0?0???0001???10110011?10?11?0??01?101010?
Microvenator_celer ??
???{12}
0????11?1101100?????00?00????????????????????0???????2??2???????????????1101?110???????0010?01?1?1
02??11000?001001000??0?10?0????????????1?11??00??2??????????????????????2001????00?0?0?010110??02
?0010000?000?0???????0???0?????????????2???0010?00001000?1001101001???1010000?00?0010100?0101??
?20????????0??????????1??11?101210100000100??000???11?001??0010000000120001101????????????????????
??????0?
Monolophosaurus_jiangi ??001000100101?10000?00?01110001000?0000??0011110001002011000000001111?
?11100??00100000000?000110011000?0011000001??010????????0000?????01???00?????1010100?100000??
?????????????0?0011110??0000?00?100?01001????0100211?1010000002000?00000000000?010100??1101?1
0?1?100?01011?00010001010100000011?0000?1001003000?0?????0?02????0?????0????????????????????????
???0100100000100100?0?0100001
000000000???0101000100100?00110?100?0??
????????????????????????
Mononykus_olecranus ??
??1100?????????????1001011??0??1?
???1?212?1??0??????10?0???????0????1010?11????121101
011020010000??101???1010??2??1?????0121?0?1??????????????????110???001000?01?10100002300001011
1?0021000111011?1010111110110110022111 2??111011?120?0?0?????????????????1?00111??00?30??????1?
??00?0?0000????????0??10011112?2001001011100001101111 0011?11???1011??1101101001?0011000102?1
1000?010100?
Neovenator_salerii ??00?010100001010000010000?110001001100010100010111 0???????????????????????????
????????????????????????0???????????????????????1??
???1?100??000??00???00??????????????????102100000?100100000000010?010100001111011111100001011?

0001000200000100011011011100100?010??????000100000100010?10??100100???????0001101?101010??
??0110100??010001000??000001000??1101010
010101000010000001100000?0001012000110011001010101011111001000110100 1????100????01??????0
00?01?10002001000?
Nothronychus_graffami ??
??
???212?????????2????20
?0?001111??????10???04?10?0?0?2010??10?0??0?000100?????00????101??00?1?00???10000110?1?111101
??100?100000?????????0?????????22????????0000000101112001 1??1???1?0??100???1100?1101000111??2
?0121?0?11102?10???0??112111100??00?000?0010110???111??1101?0??0?2000?1??0001010?0000001?011
0?0?0??0?
Nqwebasaurus_thwazi ????????2??????1000??1011?0?00??00?1???????0??????????????????????0?10?1?2011
1011002010011??0????010?0?0???????0?00?10??????????????????????????010?00?101???0000???????????
???03?11?21211?????????00?0???????10?1??01200?0??1
1111?10010??0???????0???????????1011101?10000??100?100
?010?10??0?00000010?0000001101010101221011110010101 10???????????????????????????????????1???0?0
????00???????????????????1??0??1?????0000010000010111 11?0010?0010?0?0002001110?001?0011000000 0
01000?0101010
Ornitholestes_hermanni ??00?0000?01?00101011100??10000?0110100000000?0?00111000110000001 0?100
11100??00101?01000?10000?000201000?001000000011{23}0????100000???0?0????0?0?01111101?100????
???????????????????0010?10??0000?00?100?00?011100011010001012011102101?001000000?010100??????
??10?1?101200011??1111?11?101??01001??1001010110?0??10?0?10?0020000?10?0???????????1?1?????????
?????????????010?1100001001?0??0?0?0?0?????0???????????????????????????????????1?0101??0100000?0?001
0011000000?1????010?01000010?000001?10000?0?????????????10?0?10?????????????????00??0???????????
??001??????0?100?0?0?0?0010?0?
Ornithomimus_edmontonicus ??0?????210000???11???01????0?020?????????????00???011?0111100000?0?0
??????1111112?00100?00???0??00?????010010101??011???????????0?0?11??1?00????0???000?0?0??0?0???1
110?0?10?010??10?0??10?1010?00?00??01?1?1?111??0???011??????1???????????2??????????????10??010??
1??22100???102???00??0???01??1??0?00??0?00401?00???100?0??0???0??0?01?00???0??0????????11111???
01?000002?0?????2????0??0000??1?0?0001??0?1?????0?22??0?12?00012210???010100?10001100???????10
10000?010??010????00010??10001010000?110?????00?1001?0?10?0???0??????00???0??0?0100??0001???00
1?????100?01?1?0?0?0?01000
Oviraptor_philoceratops ???100??0?11?????1????10????0?0?0??10??????0??01????1????01000???00?????1?0
101?????1???00??0??0?001?0????0??0??00???10???1?1?1??1?1?0?1????????????????????0????00???????????
????0?21???011?1111100?110?10?0?0?00???0????11??????1???????????2???????????????????????????????????
???????????1???????????0??????????????0???????????????????????10?????????1111???????????0??1??1??0???
????00?0???0????0???1????????0??????0??????0????00?0000?????0?1????????0??1???????????????????????????
????????????????????????????????????0?10?????0????????????????????????????????01????????????????????????
??
Parvicursor_remotus ??
??
??{01}1
??2?0????0???????010????????1?????0121???10??
???0????????0????????2?01?0?0??1????30???1???1??0000?0000?
???1{12}00???1?011112?200100101110000101111100011000?01?1012110110100 10????000102?11001?001
100?
Patagonykus_puertai ??
??0????????????????????1??
???0?00?0
1?0????10000????1???302011000??012100?002???????????????????????0?1000?0?????001???0??011?111002

100011??????00?10?10??????1??1112?????011?11010???????1?0???????0010010l????0211011???01001???
????????????0?0??????11120001000111000011111l1??10111??000010{12}001100110??????1????000??????
010?0?
Pelecanimimus_polyodon ??0000002100010100011101l?0?00010011100????00000?0000000111l?000??0?0?
?11201110112O2?1?01?000?0?00???????????????0?010?00???1??10???????1?00?01???????????1???01????1?
????10????0??00?001000?1??00?0?0001010??1?0??0????1030001103201?1021102010l0??10?0???101010?1
?????00010?1???1000?1?????????????????????0?????????????????????????00?110??????000???101?????1?11
000?0?200?0001?00?0100?0000011??00?1010011?1110?2210011210001121O????????????????????????????
????????????????????????????????0??
?????????????????
Piatnitzkysaurus_floresi ?????0?????????1??????????1000????110?00100?1??????????????????????????????
???????0?????????????????0?????????????????????????????0?????01???0????10??1?1?0?10?0010?010????????
??00????0?10?????????????0??????????????????????????0?1?01?00?0??0?0????0100111110?10?1010?1?0011?
???10001?1010?????11000??1?00??30?0???0???0?02????0??????????????????????????0??11?0?0?00?0?1?0?1
0?00??0??100????0??0100??0????????????010000?????????1?00????0
000?010?????11?1???0???110010?0100010?0100??0??10111???0?0????0???????????????????????????01?0???
????????
Proceratosaurus_bradleyi ??10??00000001110100110011100O?001110000???0??1???010?001100010000?????
01?0????01?????????????00??0??????0110?0000???112?0??0??0?000110101??011????????????????0?????????
?????????0?001?000?00001000?100000?1111100??0???0101000010210l?0010000000010100?0????????????
???
???
???
?????
Rahonavis_ostromi ??????????0???????????0???
???
???01201110
0000?10010010010?041001?000??10120010010020??????1?1112??????????????1100110??????2????????100
01010000????????????????????????????00??????01000?210111100111000l0000?011100211??00111??00112
1000001112101101000l1112?2011000010000010111111001110010010001200011010011001101111010l110
?001010?
Rinchenia_mongoliensis ???1?????011100?0?00??1??????????????0?????????01????1????0100?010?0?10?????
101?0????0?00??????0??0??????00?000?00?????????1???1??1???????????0???????????0?????????????????????
?????21?0?011?11111?0???0?10?0?0?0?0??0???111??????1???????????2?????????????????????????????????
?????????????????????????????????????0???????????????????????1??????????????11??????????0??1?0?0?0????
??????0???0?????????1????????????????????????00?000??????102???111?01?1????????????????????????????
???????????????????????????????????0?????????0????????????????????????????????0???????????????????0?????
?
Sapeornis_chaoyangensis ??0000000?010??1?1??0???????0??????1?00?????00001000??????11?002?00????12
?0????10?11???0??100?0?0000?0?0???
??????10??001001010?00?0?0?00?0?1?101??0??0010100?100?011?2120?2??????????0?0??????0????????????2?
?????211???0?????0????????????????05??01??0?210?0??1?0000??????0????????????111??2010??11001101111
012?001?011100?10000001111100010101111?1022??0200100100000001000?11010????1?1??010000?0?100?
3110101101?01010?1000101101001101O?11112?2?11??00100?0?????????????1????????11??1001???1110001
0??0?10??10?0?0??000?
Saurornithoides_mongoliensis ??1000??1100000??10?01?0????0???0?01???01000???0?????????1???????????
???110???????????????0?????0?00??0??????????????????????1?01????00?11?????????1?????????????0?0??0??????
???0??11?0?000?0?100000??011??0?0?????????????1?????0?000??02101?001000100010101O?0???????????????
?????????1???0?00?????0000?00??0?000??0?010?000???001????00???
???0???

01?1???0??11023001020000?0???1111111011???01???????????1?
??0???1???1??
Saurornitholestes_langstoni ???????????????????????????????????????1??????0??????????1?????????????????
?????????1?11?1??????????????????????????????1???????????????????????????????????????0???????????????
0????1??00??0?????????0????0????????????1????1???0???0??0?1000000?000100????1111010?1?1??200011?
11?10?12??00??????1??101110110?411010100????1??0???1??2?01?1????1??1????????20???????????????0??
???1??00?0?00?1?000??1?0?00?010??1111022?002???00100000?10?10210110100??1?0010011100100??????
????????0???1????0?0??0??????????????????????????01011111???101??1?01100110001100101?0011?10?10
?001?0?001?10?
Segnosaurus_galboensis ??
??1????????????????????
0010?????1?1??1??101??0?0?1?000??0???0????????0???001?0?10111?00101???????????????????????????2?
?????????????????????????????1??0???2???????1???1??11?0?0??1101
10?????????????????????????????22???????00???????1011110011102010000101000?01000?21010????01000
011110?1?102?101??0???????????????0?0??1??0???????11???11?????0??10000????001010??00000??0110200
1????
Shenzhousaurus_orientalis ??00000021000001000??1011?0?000?0011000????0000010001?????1???????0?0?
?1120111?1????0?00??0?0?????00?0?0???0?01????0????0???1???0?????????????????????????????0?????????
?????????0?001?001000010?00?0?000??1?1?0????0?????1??????1???1?????11001001??00?0???????????????
??0?????????0?00??200?0??????00?0001?0411??0??????000?0000?0??0???0??0?01?????????????????????????
???????????????????????????????????????0110?221???1210001011001000?100010101000???1000100?00010
10010100?1?010100000000010000????1????001100??00100???
?????????????????
Shuvuuia_deserti ??000000110000010101?101102100000?11000????000000000011000?11001?1?100??12201
1101120200000?10000?001000?0100000000??10???01?120000002101001??010?011111?00100?0010???110
01011110001100?001000001000?0?0000?0010011110??001????0??03201?212110202?10??10?0??10???10?1
0100211110?11210?101?0???0???0??100??01?105020?1?0010012100110100200101????1110110???001000?
0110000000230000101?1?002????110?11?10101111?01101??0221112???1101121201000?10020??01110210
12001111000?30???1??11?00000?0000????22000?010011112?200100101???0??1???101?001??????10111211
0110100100011000102?11000?011100?
Sinornithoides_youngi ????????1?000????????????1???0?00???????????????00???????????????01???????????????
??0?????????????????0??????10?????????0??0????????????????1????
000??00??000??0110?0????????????????????0?000??0?101???1??0100?10?010?????????10????0??101???????
??1?????????????????????????0?????0???10??1??01??1??0??1?0????1?12???????1?20110???00?1??001????????2
?0?10??????????????10?0010?01??????22??02????0?0000???????????????001?1????????0?1???0?0??????01???00
?1210??00?0210?0??1????????1?011???????00?????????00??????0???0????????????0100011?0?10????011?101
010?
Sinornithosaurus_millenii ?000??001?000??1111?1000??0?00001?11??01???0?00010????0000000?010?0?0??
1??0100??10111?1000100?0?001201101?0?0??0??0010???????????0??????????????0????????????????????????
?????????0?101?000?00000?00?0?0?01010??0?0??1????101010000?10010?1?00000?000100?0?????????????
??????????11???1?????????????????????????04????0????????1??0??????2???????????????00111?1120110???10?1
01110?????????????0?????????0????0000???1??????1?22??02?2?00?0000??????0?1???11??01?0????????0?11??0?
211??????1???011121????011211010?1????????????1??????????????????????????????????0??1001???001?00???1
0??1?0???1?001?10?
Sinosauropteryx_prima
000000000001?001??0?0?????0?0000????0?0????0000???????????100????00?0????1100????11?????????0??0
?00???????00??00012?0?10???????????0?????????????????????0????????0???????????????????0?001?000?00?0??
0??0?0?0?????????????????10100?1002101?0?1000000?000100?0??????010?1?1?0?10?10??11??0?20??100??
??????10?01???03????????000?0?000??1120?10000?1?1110?????????00010?111010000200?00?00????01?0??
00000?1100?0111101?1110?22??121110100010001000?1000???0???0??0?001020?000?111?????1???000100

000011?100?0??1??01?0001??0??0?1????0?01??10?00?0???0??0?00?20001101001?001100??00?0??0020??0
00?
Sinovenator_changii ??1???0001010??0010?1100111000?11???000??????000???011????????0?????????????
???0????0?000???????01????????00??12??011??????????0?0?00100????00?0??0100?110??0?10???11001010
?001??1?0??0???000??011100?0??0?1?0?0??????0101?00??0?2?1?0?10?010??1?01?0??????????10???0?210?
10??01???00??????00?01??100???0?1?401?00???10??1??01??2??0?????????????1????????20110???10?101???
??????????????????????0???10?001??????????????????????0000???????????111????1???????0?11?00?211??01
0????0011210??0011210010?10?01?11110110?0?11?00??11?111?0011???1000?10??0001???001??????00?10
???1?1?1???1??
Sinraptor_dongi ??001000000000?10000000?010?000110110000?0000001000000200000000100101??11210
???1100110000?01001001100?00010000010?0113000001000010000101?00000?10?0010100010010???10?
?0?0??0?00?1011000??0000?010100?0111010000??010101010000002100?00000000000?010100110111010?
10100001011?0011001101010101001100001?0100300000?????0002?00?0??1?1001???1??1??10????000???
??1010100?0??????0????????????????????????????0??11101210?????1000000100100101001???000?0??1000
0100000000110 0??110100001000000011010000?00010?1000110011001000101010111?000000?00?00000010
00011000?0011000000101 0?0100?000?
Struthiomimus_altus ??0???0021000??1?10?110?110?0000011?000?????0000100?11???1110?00010?0??11?0
1111?12000?0010100?0??000?0?0010010112?1011??01?0???1??0????01000????0??0000??0???0?010??110?
0?100010?000?0001001011000?00??0101?1?1010?0???011??????1???????????2??????????????10??010??????
2100???002???000?????0???1??0000??0?00401?00???100?0??00??0??1?01?0????0?10????????111110??11?0
0000200100012000010000000001?0?000101001111110022100112?0001121001001 01???10001100?10????101
0000000100100001?000010001000101000 0?110?01?00011001?0010?0?000???1100010?0?10?0100?200011??
00101???100?01011010?0001010
Suchomimus_tenerensis ???101201????0?0?????1?0?111???0?01?001000?01?????????????????000??0???????
0????100????????000?????0??1??????????????10????????0???
????????110?????0?????????????1??????????????3???00022000?01?1?2????0???0?1?01??1?10???0??????11??
0?1?0?1?1??10????11000001?01?0????????????0???????0??0???0????0100??????????0??0??0?0????00101010
2?1??0??21001??10??????????????????100???0?????0100????0?0?10000?????1??0?01?0????0?0???00?????0?
{01}{01}00?0????00?11?10??0???01011?1001?010?0??0??0??10111???1?1??0?00????110001??????????????
????????????????
Syntarsus_rhodesiensis ???0?0211?0??000???0???0000?0101001?1110??1010?001001?11?000000000?0??12
2?0??00120100?????0000?00?00??0110????00??010??00??1?0000?0???01???100??0?0?0?0?0?10000??0010?
???0??????????1?100???0011?100??10101000??????????1010?010?010??00000??1??00??00?0110??0?1100?0
0???001000?000001 0002??100010?00??0?00?03010???1?0000020?000?00001?1?0?1??00?????????0??1000?
1000?0?1?0?10001100??1???0??0?0??1??000001?001000110?0?11110000???011000?0000????1000?000110
1???0001?0?00???00011011000101100 10000?0?110000??00000{01}010000101?10110?000?10?1010??1001
000000?10?0?100?0??000????1001000?
Tanycolagreus_topwilsoni ??1??000?00?00????????????????????????????1?0000???0??????????00000?1??12
201?00?????????0?00?110?1??????????????000?010???
?????????????????????????????????1000????101??01??0?10??0??000?0?????????????011???1??0???1????????
??????0?0?00100????1001100?001?0?0?????????00?0???????20?00????1??????????????00010?1?10000??101
110?011?0111000000010??11000010100?110002210?20?100000000???????????????????????????????????111
101?1????0??????????????????1010110001100010010 0000?010111?0010001100 0100?2000111100100010?0
?00010?0002001000?
Tarbosaurus_bataar ??0???0000001101 0000?100110?000000??0000????1100101001 20110011000?111101???
100001??01000??0?1010?112?111011001010??0103100001 0000?000??01?10010?1?000001001??0000??????
?0??????0??0011?00??0000?00?100001?111101110010101010000 10200??0?0000000?00010???????????????
??0??0??????
?????0?????????????????????????????0?????????32??

??1?????1??????1????
??000?0?
Tawa_hallae ??0001201000??00????01001?11000?00000?1????0?000?0011?200?001000010?0??12100???11
00??0????000?0?00?0?0???11010000??0????????????0?020?001???000??????000??1??0??????1????????????
?10??000?00000?00?100?00??0????0????????00??00002000?0000?0000?000100?0???????1100?00??00?1???
??0?0?????????????1????1???0???????0???????0??00????0?0?00?????????????????????????1?10100?01001100
00?????????????0??1????000??10??00?01110001110?000000000000?0???0???0?00??00000100?000?0110????
????10??????????????????????001??2000000??100????????????????????????0001000000000?010??00?00???0
0?1???????
Torvosaurus_tanneri ??001010???????0??????????1100????00000101??????????1?2??10000???0101??1100??
?00????????????0010?00??????????????0?1?0?0???
??????0?0????????0????????????????????????00??0??0?0001?00?0????1????010000???1?10?1000?1?1?11????
10002?10100????11000??1?0??03??????????1?02??0?0??0?1??????11?0??????????0??0?0000??????2?0??020
1?00?0200?1?01001?????01111?0?10?01?????0????10????00100110??0????00?0?000000?????000?0?00???0
000000100?10?00?1?0?0????0??1????0?010?0??0??0??00111???0?0????00???011000111??????????????01?0?
??????????
Troodon_formosus ??0???001?0?0?010?011100?10?0000?1??0??1??????00???0????????00????110??????100
??0??00?00?01??00??01??0?0???0?0?1???01030??1?11??0?0011000???001?010?1000001???010?1110?10?10
?111??1?1??010?100??0110?00????????0????????101?00?00?201?0?1000220?001010?????111?10?111??210
110?01?10?100101??????1??1011??0?10501?10?0????011?0?0?20?0??1??????????2???????????????1?00110??
?0?1?0?10??01?0?????????0????000010?0???????220??????001??????????0?????????????????????????????010?
?0001???0??11??23??1?21000?0?0001?11120110?0?1???0?00110101??1????1?0100012000110?001?????100
111?1?011?1??010?
Tsaagan_mangas ??00000020000?011010?100110?000000010001???00000100?1?0001000101010?0??1100??
???0?11100001100?0?001200?000010?000?00103????0?000000100101001000?11010101100???0000??10110
?1000000?001?000?00000?00?0?0?010011101011111?010101?0002101?0?1000000?000100???????????????
00?0?????110??1?
???
???
?????
Tyrannosaurus_rex ??10?000000011010101110011?0?000000010000010111011010002011001100011111?0111
0110?1111010000?011?1001120111011001010100103?00001000000100101?10?00?100000010010?0?010?0
10??0?10000000000110000?00000?00?1000010111?011100101010100001020000000000000000000010100000111
010?11?00000011?00010001000000000000100000010010031000000?0100000100000020000000?100110???101
??00010010100000001100010012?00010001001001?0?0000101000????03200021??00000?00010010101110000
0000?1200011000000101011000101000010000000102100101011012001210010001000100010111?0010?0??
0?0100020001101001?0011100001?110002000000?
Unenlagia_comahuensis ???
???
???0?20
?1100????10011011011??4000?01?0???????????????????????10???????????????????1?00110?010011021??????
???01000?2100101001110001000101011101110101011?010?1
110210011????110100010010?1110000100??????????????1???
?
Velociraptor_mongoliensis ??00000020000?01111011001110?000000110001???00000100011000 0?00101010?
0??1120100?10?11101100100?0?000?0???00?10?000?0010?1?1?0100?00??0010?001?00?1?01001?000??0?0
0???11110?100000010011000?00000?00?0?000100?1?0?0111101010?110000?001100100000000000100?0??1
11?010????012000????10??01???1?00??0?1001011?01?00501010?0010001??010?1????01?110??1?12001100
1020110??100?111010?1?0??01?0010??1100??0?1?10000???010???0?220002?2?0010000001000?110011100

101?00?1000?0110002110?01011?0001111022001021010?0111??1?11?11?000010000010?1???100???0????0
000?20001???101?0011110010001111?001010?
Zanabazar_junior ??1000001100000101010100111000000111000 1????00001000??1???1???????????????111
?????00?00?0100?0?00120100?0000?0???????????????????0011000????01?0??110001010?0010???10????0??
1110???0?001000000?0110?000???????????1100?101000110210 1?0010001000101010?0??????????????????
??????????????????????????????????401010?00???0??00???2??0?????????1?12???????????????????????????
???
????1??????0???20001?????1?????????????1?????????
??
Zuolong_salleei ??00??00?00?010101001?0?0?0?0000001110000000??????00???????0??010?0?0??1?101110
11????00?0?00000?0?1100?00?1000000010113 00??0????0??
???10100001020?0000100???????010100???????10?0?1011000 11??
0?10??00?01???????????????????40001?000??000000??0?00????????????????????????????1?1?1???010001?
??010???????00?000???????????????????????????????0???00100??????????00?0???0???100?0000011?00101
12?00????????????????????1?0110001000010010000010 10111?0???011??000????????????010????100010101
000????0???
Aepyornithomimus_tugrikinensis ??
???
???
???
???
???
????????????????????????000???10111001?1???100001?01011?0010010
